# Bias in Ligation-Based Small RNA Sequencing Library Construction Is Determined by Adaptor and RNA Structure

**DOI:** 10.1371/journal.pone.0126049

**Published:** 2015-05-05

**Authors:** Ryan T. Fuchs, Zhiyi Sun, Fanglei Zhuang, G. Brett Robb

**Affiliations:** RNA Research Division, New England Biolabs Incorporated, Ipswich, Massachusetts, United States of America; East Carolina University, UNITED STATES

## Abstract

High-throughput sequencing (HTS) has become a powerful tool for the detection of and sequence characterization of microRNAs (miRNA) and other small RNAs (sRNA). Unfortunately, the use of HTS data to determine the relative quantity of different miRNAs in a sample has been shown to be inconsistent with quantitative PCR and Northern Blot results. Several recent studies have concluded that the major contributor to this inconsistency is bias introduced during the construction of sRNA libraries for HTS and that the bias is primarily derived from the adaptor ligation steps, specifically where single stranded adaptors are sequentially ligated to the 3’ and 5’-end of sRNAs using T4 RNA ligases. In this study we investigated the effects of ligation bias by using a pool of randomized ligation substrates, defined mixtures of miRNA sequences and several combinations of adaptors in HTS library construction. We show that like the 3’ adaptor ligation step, the 5’ adaptor ligation is also biased, not because of primary sequence, but instead due to secondary structures of the two ligation substrates. We find that multiple secondary structural factors influence final representation in HTS results. Our results provide insight about the nature of ligation bias and allowed us to design adaptors that reduce ligation bias and produce HTS results that more accurately reflect the actual concentrations of miRNAs in the defined starting material.

## Introduction

The amount of small RNA (sRNA) research has increased dramatically in recent years as the importance of these RNAs has become appreciated. Eukaryotic regulatory sRNAs typically range in size from ~20 to 30 nt and the three major classes are microRNAs (miRNA), small interfering RNAs (siRNA), and piwi-interacting RNAs (piRNA). Although these classes differ in their biogenesis and the targets of their regulatory effects, they all share the ability to cause gene silencing through an anti-sense base-pairing recognition pathway (reviewed in [1]). miRNAs in particular have been implicated in the regulation of a wide range of cellular processes including maintenance of pluripotency and cell differentiation [2]. This combined with the fact that altered miRNA expression profiles have been implicated in a number of disease states [3–5] highlights the importance of miRNAs in biology and the need for continued development of research tools for sRNAs in general.

High-throughput sequencing (HTS) is a powerful tool for the analysis of sRNA molecules [6,7]. HTS allows the detection of single base differences between molecules, the discovery of unknown molecules and the determination of the differences in sRNA composition or expression between different samples. For sRNA analysis, libraries are typically constructed through a multistep process starting with the ligation of a pre-adenylated DNA adaptor (AppDNA) to the 3’-end of the sRNAs using a truncated version of T4 RNA ligase 2 (T4 Rnl2tr). The products of this reaction are then subjected to the ligation of an RNA adaptor to their 5’-end using T4 RNA ligase 1 (T4 Rnl1). After this ligation, the sRNA sequence is now flanked by two defined adaptor sequences and can be easily reverse transcribed into cDNA and amplified by PCR prior to HTS sequencing.

While HTS has many advantages over low-throughput sRNA analysis techniques such as quantitative PCR (qPCR) and Northern blotting, it also suffers from disadvantages such as the cost per sequencing run and the extensive processing steps required to convert a sample into a library for sequencing. These processing steps, particularly the ligation steps, have been shown by several recent studies to introduce bias in the results [8–14]. Previous studies have investigated the effects of both 3’ and 5’ ligation steps and have suggested that randomizing the adaptor sequences close to the ligation junction reduces ligation bias and improves HTS results [11,14,15]. In our previous work, which investigated 3’ adaptor ligation in isolation, we also made the same suggestion [9]. However, it remained unclear whether this approach actually generates HTS results that better reflect the contents of the sRNA sample because the sRNA sample in previous randomized adaptor studies was usually either an undefined random pool or a defined pool of low complexity. Also, it was not known which sequences in a randomized adaptor pool were being ligated to a particular RNA or whether the randomized region absolutely had to flank the ligation junction to see a reduction in ligation bias. In this study, we not only continue our previous work by investigating 5’ adaptor ligation in isolation, but we also seek to address some of the lingering questions about randomized adaptors by using complex, defined pools of 50 and 962 unique miRNA sequences and subjecting them to library construction and HTS sequencing. The objective is to determine the accuracy of HTS results relative to the starting miRNA pool when using different combinations of 3’ and 5’ adaptor pools.

Our results show that HTS results better reflect the starting sRNA pool when randomized adaptors are used in library construction. Improvement is seen whether the randomized adaptor is used in 3’-end ligation or 5’-end ligation and the most improvement is seen when randomized adaptors are used in both ligation steps. Furthermore, the randomized region does not have to be adjacent to the ligation junction, which indicates that primary sequence at the junction does not have a large effect. We also observe that miRNAs preferentially ligate to adaptors in the randomized pool with which they have the potential to form particular structures. The use of a 5’ adaptor that has a region complementary to the miRNA-3’ adaptor hybrid can promote the formation of structures favorable for ligation and also reduce bias. These observations support our conclusion that structures within the sRNAs and between the sRNAs and adaptors are the most important determinants of ligation efficiency with T4 RNA ligases. Finally, we present a new approach for designing adaptors for small RNA library construction. Adaptors are designed to contain internal randomized regions and complementary regions that together induce favorable ligation structures. This method substantially reduces representation bias in sRNA libraries as compared to the single sequence standard adaptor method, and allows for precise determination of ligated RNA ends.

## Materials and Methods

### Oligonucleotides, library preparation and sequencing

The miRXplore Universal Reference pool of 963 miRNA oligos, which comprises 962 unique sequences, was purchased from Miltenyi Biotec Inc (Auburn, CA). The pool contains all mature human, mouse, rat, and related viral miRNAs as published in miRBase release 9.2 [16–19]. Custom RNA and DNA oligonucleotides were synthesized by Integrated DNA Technologies (IDT, Corralville, IA). The sequences are listed in S1 Table. A special order from Thermo Scientific Molecular Biology (Dharmacon) was fulfilled to produce a random RNA oligo pool for 5’-ligation testing. After synthesis of the 21 base random region the bulk of the oligos were removed. Then synthesis resumed so that the 5’ constant region could be added. This ensured that the initial composition of the randomized region was the same for the two oligo pools. DNA adaptors were adenylated using a 5’-DNA Adenylation Kit (New England Biolabs, NEB, Ipswich, MA) according to the instructions provided by the manufacturer. Adenylated products were separated from unadenylated products on 20% Tris-borate-EDTA (TBE)-urea acrylamide gels. Bands corresponding to adenylated products were isolated, crushed and soaked in 10 mM Tris-HCl pH 8.0 overnight at room temperature with constant rotation. The eluted, adenylated products were then recovered by precipitation with ethanol.

Library preparation for high throughput sequencing was carried out using protocols and reagents found in the NEBNext Small RNA Library Prep Set for Illumina (NEB). The components of the kit, specifically adaptor and primer sequences, were substituted to meet the needs of this study, as indicated in the figures and S1 Table. PCR products from library construction were gel purified on 6% acrylamide gels, passively eluted, recovered by ethanol precipitation, and then analyzed for purity on an Agilent 2100 Bioanalyzer (Agilent Biotechnologies, Santa Clara, CA) using an Agilent DNA 1000 Kit. The resulting purified libraries were sequenced on the Ion PGM sequencer (Life Technologies, Carlsbad, CA) using Ion 316 chips and the Ion OneTouch for bead templating or sequenced on the Illumina MiSeq instrument, as indicated.

### Bioinformatics

Sequencing reads from the Ion PGM and Illumina MiSeq runs were imported into Galaxy ([20–22]; http://galaxyproject.org/) where tools were used to remove the adaptor sequences. Sequences from experiments involving synthetic miRNA oligonucleotide mixtures were annotated in Genomics Workbench 5.1 (CLC bio, Cambridge, MA) by using a reference file containing the sequences found in each mixture and a zero mismatch tolerance. Each sequencing run produced at least 143,900 annotated reads, which equates to a minimum of 149-fold more reads than unique sequences present in the miRXplore Universal Reference. The number of annotated reads was divided by the number of unique sequences present in the oligonucleotide mix that was used in library construction in order to produce a “normalization factor”. The number of reads for each miRNA sequence was then divided by the normalization factor to yield a normalized reads value. The normalized reads value was then divided by an expected normalized reads value and graphed. If an experimental replicate of a library was made, then the normalized reads values were averaged before graphing. The expected normalized reads value was generated for each miRNA based on the known abundance of the miRNA in the mixture prior to library construction.

RNA CONTRAfold [23] (http://contra.stanford.edu/contrafold/index.html) was used for secondary structure prediction of single RNA molecules using default settings. Vienna RNAcofold [24] (http://rna.tbi.univie.ac.at/cgi-bin/RNAcofold.cgi) was used for cofolding structure analysis of adaptors with ligation substrates. The default settings for the minimum free energy model were used except for that the folding temperature was set to 25°C and the 1999 Turner Model was used as the energy parameter, as it was in our previous study [9]. Cofolding structures between the miRNA sequences (molecule A) and the randomized 3’ adaptors (molecule B) were first predicted using the RNAcofold program. Only when the free energy of AB formation is smaller than that of either single molecule A or B, a secondary structure of A and B is considered. Based on the formation of stem(s) and loop(s) and the relative position of the A-B junction in the loop or stem structure, all the secondary structures of AB were classified into 16 categories (S2 Fig). The same cofold analysis was applied to the randomized 5’ adaptors (molecule A) and the miRNA-3’adaptor combined sequences (molecule B). The RNA cofold batch analysis pipeline was written in custom perl scripts that are available upon request.

The sequencing files used to generate the data presented in this publication have been deposited in NCBI's Gene Expression Omnibus [25] and are accessible through GEO Series accession number GSE67053 (http://www.ncbi.nlm.nih.gov/geo/query/acc.cgi?acc=GSE67053)

### Quantitative PCR

Several miRNAs in the oligonucleotide mixtures were analyzed by quantitative PCR (qPCR). TaqMan probes and primer sets (Life Technologies) were purchased for the miRNAs of interest and an RNA oligo matching the miRNA sequence was purchased from IDT to be used as a standard. Target RNAs were reverse transcribed using a TaqMan MicroRNA Reverse Transcription Kit (Life Technologies) according to the instructions provided by the manufacturer. The resulting cDNA was used as a template for qPCR reactions using TaqMan Universal PCR Master Mix (Life Technologies). The qPCR reactions were run on a Bio-Rad (Hercules, CA) CFX384 Real-Time System and results analyzed with Bio-Rad CFX Manager software.

### Ligation reactions

Experiments determining the ligation efficiency of RNA adaptors to the 5’-end of defined substrates were performed as described below. Mixed RNA/DNA oligos were purchased from IDT that correspond to a miRNA sequence on the 5’ half of the oligo and the DNA sequence of the 3’ A1 adaptor on the 3’ half of the oligo (S1 Table). Each ligation experiment contained one of these oligos (0.4 μM), a 5’ RNA adaptor (0.83 μM), 1.7 units/μl of RNase Inhibitor, Murine (NEB), and 0.8 units/μl of T4 Rnl1 (NEB) in a buffer (31 mM Tris-HCl pH 7.5, 6.3 mM MgCl2, 0.63 mM DTT, 0.2 mM ATP, 5.2% PEG8000) that mimicked the buffer conditions during the 5’-ligation step of the sequencing library protocol. The ligation reaction was incubated at 25°C for 1 h and then the products of the reaction were analyzed on an Agilent 2100 Bioanalyzer (Agilent Biotechnologies) using an Agilent Small RNA Kit.

## Results

### Bias in small RNA library construction measured by high throughput sequencing of a defined pool of 962 miRNAs

The use of a defined mixture of small RNAs allowed us to directly compare the results of different library preparation conditions to an expected result. We used a commercially available mixture (miRXplore Universal Reference (URef); Miltenyi Biotec Inc.), which contains 963 miRNAs (962 unique sequences) mixed in equal concentration.

**Fig 1:**
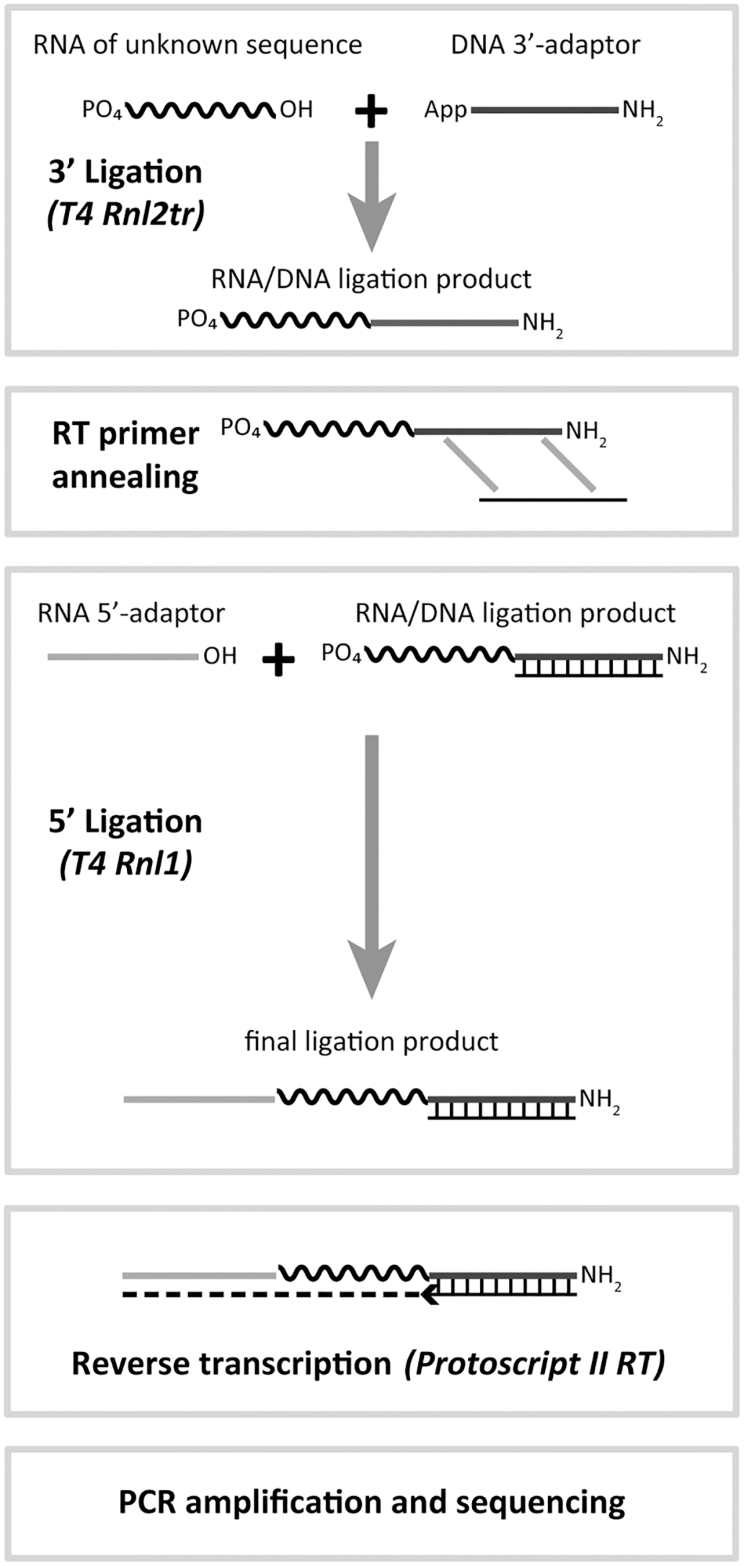
Typical HTS library construction scheme for miRNAs. An RNA sample is subjected to a ligation reaction to attach a 3’ adaptor sequence. This reaction contains T4 RNA ligase 2, truncated (T4 Rnl2tr) or T4 RNA ligase 2, truncated KQ (T4 Rnl2trKQ) and an adenylated DNA adaptor. Next a primer for reverse transcription is annealed to the 3’ adaptor. The primer will also anneal to excess unligated 3’ adaptor, which will reduce undesired products in the next ligation step. After primer annealing, the natural presence of a phosphate group on the 5’-end of miRNAs is exploited as a substrate to ligate an RNA adaptor to the 5’-end in the presence of T4 RNA ligase 1 (T4 Rnl1) and ATP. The final ligation products containing miRNA sequences flanked by two adaptor sequences are reverse transcribed and PCR amplified in order to introduce the primer regions needed for HTS.

**Fig 2:**
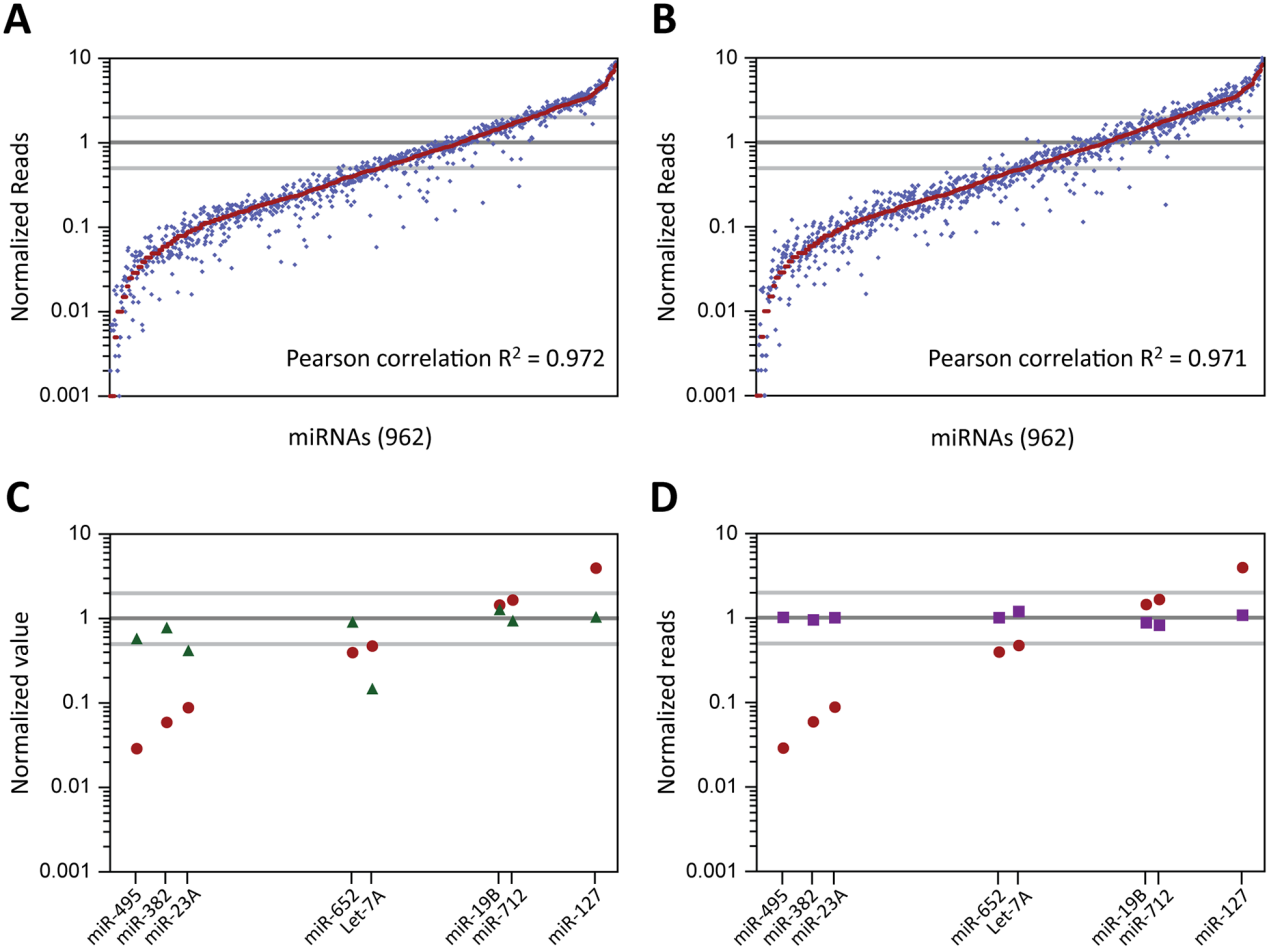
HTS results from libraries made with the miRXplore Universal Reference. (A) Comparison of a library constructed with 3’ A1 and 5’ A1 adaptors (red) and a second sequencing run of the same library (blue, sequencing replicate). The miRNAs were arranged in order (left to right) on the x-axis according to their normalized reads in the first data set (low to high). All miRNAs were expected to have a normalized reads value of 1 (dark gray line). The interval of 2-fold from the expected value (equivalent to a 2-fold over or under representation) is shown with light gray lines. (B) Same as (A) except that the results plotted in blue are from a separate library that was made using the same method as the first library (library construction replicate). (C) Comparison of HTS and quantitative PCR results. The data points for the abundance of 8 miRNAs according to HTS are plotted at the same locations on the x-axis and y-axis (normalized reads) as they were in the graphs in (A) (red circles). The data points for each miRNA according to quantitative PCR analysis (normalized concentration) are represented by green triangles. (D) Comparison to HTS results using a synthetic cDNA pool. The data points for the abundance of 8 miRNAs according to HTS are plotted at the same locations on the x-axis and y-axis as they were in the graphs in (A) (red circles). The data points for the abundance of the 8 miRNAs when an equimolar pool of 8 synthetic cDNA oligos was PCR amplified and subjected to HTS are shown as violet squares.

The URef pool was first subjected to a library construction protocol with 3’ A1 and 5’ A1 adaptors (sequences in S1 Table) as outlined in Fig 1 to observe the extent of bias in HTS results. The library was sequenced on the Ion PGM, the sequencing reads were annotated with a zero mismatch tolerance, counted, and normalized such that each miRNA was expected to have a normalized read value of ‘1’. These normalized read values are plotted from highest to lowest in red on a logarithmic scale in Fig 2. The graph shows that a minority of the miRNAs were within 2-fold of the expected normalized value and that there is ~4 Log10 unit (~10,000-fold) spread between the abundance of the highest versus lowest represented miRNAs. We next tested whether these results were repeatable using two approaches. These approaches were: taking a second aliquot of the original library for a new sequencing run (sequencing replicate) and repeating the entire library construction protocol to generate another library (library construction replicate). The normalized sequencing results for the former approach are plotted in blue in Fig 2A and the results for the latter approach are plotted in blue in Fig 2B. Both approaches show that the normalized read values for the miRNAs are highly repeatable with Pearson correlation R^2^ values to the original data set of 0.972 and 0.971, respectively. There are miRNAs that showed normalized reads variability of over 2-fold between the original data set and the additional data sets, but less than 8% of miRNAs had a discrepancy of 2-fold or more. On the whole, these results show that there is significant bias in the sequencing results from libraries constructed with 3’ A1 + 5’ A1 adaptors and that the bias is repeatable.

We performed a qPCR assay using TaqMan probes and reagents (Life Technologies) that targeted 8 miRNAs in the URef pool to observe the extent of bias inherent in a measurement method different from HTS. The 8 miRNAs consisted of 3 miRNAs that had a low abundance according to sequencing results (miR-495, miR-382, miR-23A), 2 miRNAs that were near the median abundance (miR-652 and Let-7A), and 3 miRNAs that had a high abundance (miR-19B, miR-712, miR-127). Of the 8 miRNAs, 7 are closer to their expected equal abundance when measured by qPCR versus when analyzed by sequencing and 6 of the 8 are less than 2-fold from their expected abundance versus only 2 of 8 in the sequencing results (Fig 2C). These results support the observation that there is significant bias in the sequencing results and that the distribution of sequencing reads does not accurately reflect the composition of the miRNAs in the URef relative to each other.

The bias that is present in the sequencing results could have been introduced at one or multiple points during the process of library construction and sequencing. Previous studies have shown the variability in ligation efficiency for different miRNAs with the same adaptors and/or have implicated ligation bias during library construction as the main contributor to bias in the final results [11–15,26]. We designed an experiment to test if the protocol steps from PCR amplification onward contribute to the bias as well. Oligonucleotides were ordered with sequences that mimicked the cDNA product (reverse complement of the 5’ adaptor-miRNA-3’ adaptor sequence) that would be formed after the reverse transcription step of the library construction protocol for 8 miRNAs. The 8 miRNAs chosen were the same as those listed above for the qPCR experiments. The 8 cDNA mimic oligos were mixed in equal concentration and the rest of the library preparation and sequencing protocol was carried out from the PCR amplification step onward. The HTS results from this experiment are compared to the HTS results from the standard URef library construction protocol in Fig 2D. All 8 of the miRNAs are closer to their expected equal abundance in the cDNA mimic results and all of them are less than 1.3-fold over or under their expected value. These results suggest that the amplification steps in library construction and the downstream steps to prepare the samples for sequencing are not responsible for the bias seen in the URef HTS results.

### Absence of primary sequence bias in 5’-adaptor ligation

In order to develop a strategy to reduce bias in sRNA HTS results, we wanted to have a better understanding of the nature of the ligation bias in each ligation step. We previously used randomized oligonucleotide pools to study bias in 3’ adaptor ligation [9], and here used a similar approach to determine 5’ adaptor ligation bias (S1 Fig). Briefly, a pool of RNA oligos were synthesized such that they contained a 21 nt constant region on the 3’ half of the oligos and a 21 nt randomized region on the 5’ half. Part of the custom oligo synthesis was allowed to continue past the randomized region such that a randomized pool of oligos with a constant region on both ends was synthesized. The pool of oligonucleotides lacking a 5’ constant region was then subjected to a ligation reaction containing the 5’ A1 adaptor and T4 Rnl1. The products of this reaction and the pool of oligos with a constant region on both ends were separately reverse transcribed and PCR amplified to prepare two libraries for sequencing on the Ion PGM. The former of the two libraries was called the “output” as it represented the sequences that were captured during adaptor ligation, while the latter was called the “input” as it represented the composition of the randomized oligos without a ligation step.

The Ion PGM produced ~2.5 million sequencing reads for both the input and output libraries and the sequence content was analyzed in several ways. First, primary sequence composition at each position in the 21 nt randomized region was determined (S2 Table) and plotted in enoLOGOS format (S2 Fig, panel A; [27]). The nucleotide frequency at each position in the output library was then normalized to that of the input library and the enrichment of a nucleotide at a given position was calculated as previously described [9,28]. At every position there was less than 5% enrichment for a given nucleotide, which indicates that there is minimal preference for a particular nucleotide at a particular position in the output library (S2 Fig, panel B). This lack of primary sequence preference for libraries prepared with 5’ adaptor ligation is consistent with our previous results showing a lack of primary sequence preference for 3’ adaptor ligation [9].

### RNA secondary structure influences 5’-adaptor ligation

We used CONTRAfold to predict the secondary structure content of the RNAs in each library [23]. The RNAs were sorted into groups based on the number of unpaired bases at their 5’-end, the percentage of a group in each library was calculated, the enrichment of each group in the output library was determined by the equation ‘(output-input) / input’, and the results of this analysis are shown as a graph in S2 Fig, panel C. The results show that RNAs with zero free bases at their 5’-end are under-represented in the output library. This is consistent with our previous work that showed RNAs with paired bases at their 3’-end were under-represented after 3’ adaptor ligation [9]. Our 5’-end results are also consistent with the 3’-end results in that enrichment of RNAs in the output libraries generally increases as the number of unpaired bases increases. There is a difference in the 3’-end versus 5’-end results with RNAs that are predicted to have no secondary structure. Our previous study of 3’ adaptor ligation showed that these RNAs are neither favored nor disfavored (enrichment value ~0) when ligated with T4 Rnl1 [9], but our 5’ adaptor ligation results indicate these RNAs, denoted by ‘no pairing’, are disfavored by T4 Rnl1 (S2 Fig, panel C). It is possible that differences in the reaction substrates of 5’ adaptor ligation (RNA-3’OH and 5’ PO4-RNA) versus 3’ adaptor ligation (RNA-3’OH and 5’App-DNA) could account for this difference. However, the fact that our previous study shows that ‘no pairing’ RNAs were significantly enriched when 3’ adaptors were ligated using variants of T4 Rnl2tr while the enrichment value for T4 Rnl1 was ~0 suggests that ‘no pairing’ RNAs are not favorable substrates for T4 Rnl1 in general [9].

### RNA and adaptor cofold structures influence adaptor ligation

Using the Vienna RNAcofold algorithm [24,29,30], all of the RNA sequences captured in each library were cofolded with the 5’ A1 adaptor. The cofold structures were classified into categories based on their minimum free energy structure configuration around the ligation junction as shown in S2 Fig, panel D and as described previously [9]. The percentage of RNAs in each cofold category in the input and output libraries is shown in bar graph format in S2 Fig, panel E. Cofold categories 2, 3, 10, and 11 were not included in this graph since there were no representatives of these classes in the libraries. The enrichment of a cofold category in the output library is shown in S2 Fig, panel F and was calculated by taking the category percentages and using them in the equation ‘(output-input) / input’. The enrichment results suggest that T4 Rnl1 prefers 5’ adaptor cofold structure categories 5, 13, and 9, which represent stem loops with a break in the loop, while the other most common categories 8, 16, and 12, which represent a bulge in a stem, are less favorable for ligation. The observation that stem loops are favorable for ligation is consistent with the fact that the natural substrate of T4 Rnl1 in vivo is a break in the anticodon loop of tRNALys [31–33]. The preference for the lower abundance categories 1, 4, 15, 7, 14, and 6 is more ambiguous because these categories are represented in such small numbers that a small difference in abundance can result in a significant enrichment value even though each category continues to represent less than 2% of the overall distribution.

Together, the above results lead us to conclude that for 5’ adaptor ligation, T4 Rnl1 does not show a significant preference for substrates based on primary sequence composition, but does show preference for substrates that lack secondary structure at the 5’-end and a preference for particular adaptor-substrate cofold interactions. These general results are consistent with our previous study on 3’ adaptor ligation, but it should be noted that there are differences between the enrichment results for 5’ adaptor cofold categories and the enrichment results for 3’ adaptor cofold categories [9]. We do not find these differences surprising since the substrates are different for 5’ adaptor ligation (RNA-3’OH and 5’ PO4-RNA) versus 3’ adaptor ligation (RNA-3’OH and 5’App-DNA) and the two studies had different adaptor-constant region sequence combinations.

### Analysis of miRNA-adaptor folding in HTS results

The fact that a ~10,000-fold spread in miRNA abundance was seen in HTS results when A1 adaptors were used raised the question of what factors determined whether a given miRNA would be under-represented or over-represented in these libraries. Since our independent studies on 3’ adaptor ligation and 5’ adaptor ligation both implicated miRNA secondary structure and miRNA-adaptor cofolding as contributors to ligation efficiency, we conducted cofold analysis on the 962 URef miRNA sequences with A1 adaptors and separated the cofold results into categories as described above.

**Fig 3:**
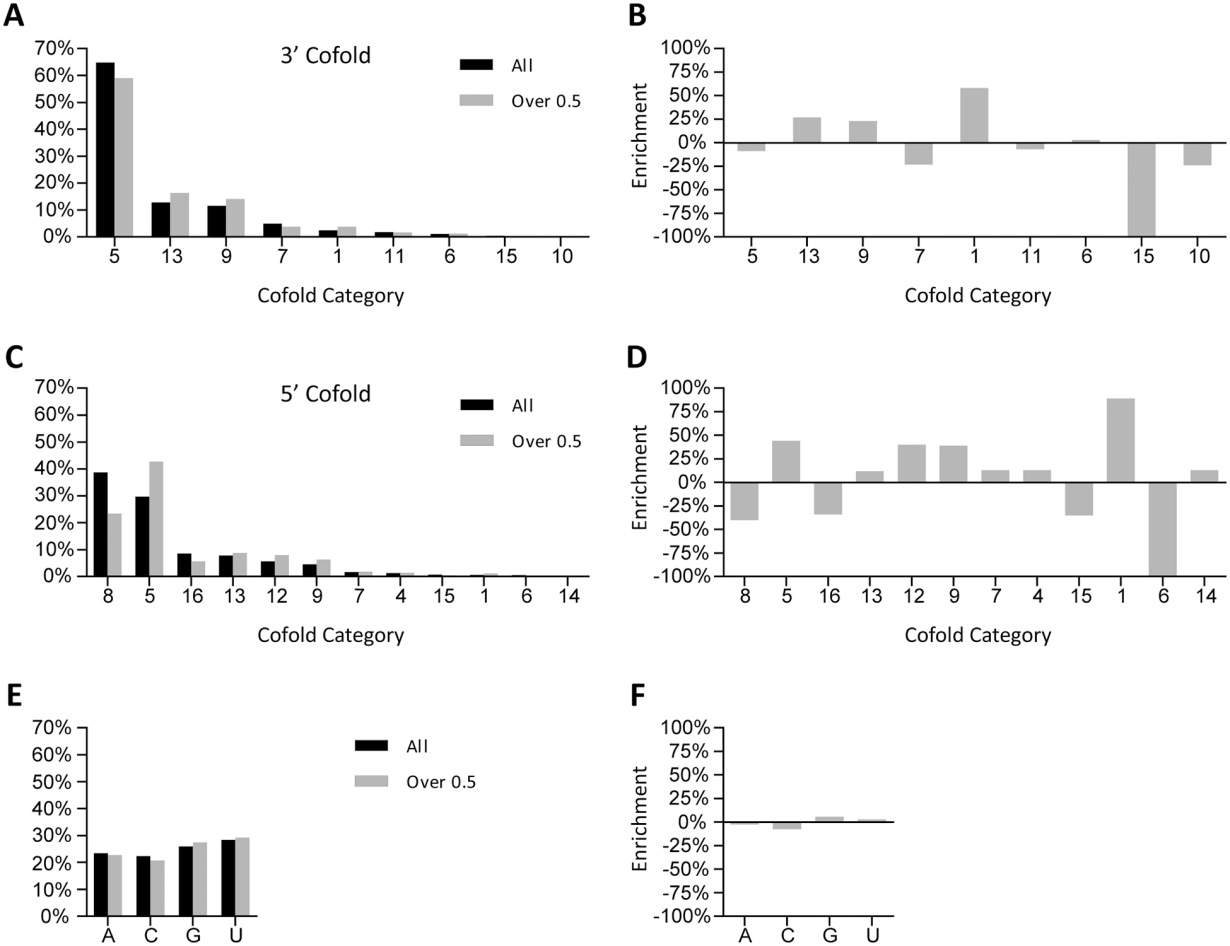
Distribution of cofold structures with 3’ A1 and 5’ A1 adaptors and the miRNA sequences in the miRXplore Universal Reference. (A) Distribution of cofold structures of miRNAs with the 3’ A1 adaptor for all 962 miRNAs (black) or the 424 miRNAs with a normalized reads value of 0.5 or higher in HTS libraries made with 3’ A1 + 5’ A1 adaptors (gray). (B) Enrichment of 3’ cofold categories for the ‘Over 0.5’ miRNAs. The enrichment value in each category was determined from the cofold distribution in (A) using the equation ‘(Over—All)/All’. A positive enrichment value indicates that the category is enriched in the ‘Over 0.5’ miRNA population, while a negative enrichment value indicates that the category is depleted in the ‘Over 0.5’ miRNA population. (C) Distribution of cofold structures for miRNA-3’ adaptor hybrids with the 5’ A1 adaptor for all 962 miRNA sequences (black) or the 424 miRNAs with a normalized reads value of 0.5 or higher in HTS libraries made with 3’ A1 + 5’ A1 adaptors (gray). (D) Enrichment of 5’ cofold categories for the ‘Over 0.5’ miRNAs. The enrichment value for each category was determined from the cofold distribution in (C) using the equation above. (E) Distribution of primary sequence composition for all 962 miRNAs (black) or the 424 miRNAs with a normalized reads value of 0.5 or higher in HTS libraries made with 3’ A1 + 5’ A1 adaptors (gray). (F) Enrichment of primary sequence composition for the ‘Over 0.5’ miRNAs. The enrichment value for each category was determined from the cofold distribution in (E) using the equation described above.

The distribution of 3’ and 5’ cofold categories for the 962 miRNA sequences is presented in Fig 3A and 3C, respectively (black bars). In addition the distributions for the 424 highest represented miRNA sequences, which are those that have a normalized reads value of greater than 0.5 in the A1 adaptor HTS results, are also shown in Fig 3A and 3C (gray bars). The enrichment of each category in the ‘Over 0.5’ data set was calculated using the equation ‘Over 0.5—All / All’ and the results of the calculations are shown in Fig 3B and 3D for 3’ cofold and 5’ cofold, respectively. The enrichment results show cofold categories that are enriched or depleted in the ‘Over 0.5’ miRNAs population. This is in contrast to enrichment of primary sequence content, which is minimal (Fig 3E and 3F). The enrichment of many cofold categories differs from what were observed as positively or negatively enriched in our studies of isolated 3’ adaptor [9] and 5’ adaptor ligation (S2 Fig). This is likely due to the fact that the miRNA substrates in the URef do not have an appended constant region that would influence the results of cofold analysis and introduce constraints into what structures can be formed with a given adaptor.

### Influence of individual RNA secondary structure and RNA-adaptor folding attributes on HTS results

The 3’ CONTRAfold, 5’ CONTRAfold, 3’ cofold, and 5’ cofold analyses provide 4 different folding attributes for each miRNA in the URef pool. We wondered whether these 4 attributes would help explain the relative representation of the miRNAs in sequencing results from libraries constructed with 3’ A1 + 5’ A1 adaptors. To test this we took the 3’ and 5’ cofold enrichment results from the previous section, the 3’ CONTRAfold enrichment results from our previous work [9], and the 5’ CONTRAfold enrichment results from S2 Fig, panel C and classified each category from these analyses as ‘unfavorable’ for ligation if the enrichment value was -10% or worse. An attribute was considered ‘favorable’ if it had an enrichment value of +20% or higher (3’ cofold, 5’ cofold) or +5% or higher (3’CONTRAfold, 5’ CONTRAfold). We then took the HTS results from libraries constructed with 3’ A1 + 5’ A1 adaptors and separated the data into groups based on whether a miRNA sequence had an ‘unfavorable’ or ‘favorable’ classification for an attribute. The results of the individual attribute analysis are presented in S3 Fig as individual points. The graphs show that 3’ cofold, 5’ cofold and 5’ CONTRAfold influence the representation of miRNAs in sequencing significantly while 3’ CONTRAfold has a modest effect. These results suggest that miRNA-adaptor cofold has a bigger effect on miRNA representation in HTS results than miRNA secondary structure and that the cofold for the 3’ adaptor ligation and 5’ adaptor ligation both contribute.

### Adaptors with internal regions of randomized sequence reduce bias in small RNA sequencing results

Previous studies have shown that a pool of adaptors with regions of randomized sequence adjacent to the ligation junctions can help make the representation of sRNAs in HTS results more diverse [11,14,15]. Our results above and in our previous work [9] suggest that the randomized adaptors may produce more diversity by providing flexibility to make favorable ligation substrates. We tested different combinations of adaptors with randomized regions for the ability to reduce the bias in URef sequencing results. We designed 3 randomized adaptors, two 3’ adaptors and one 5’ adaptor (sequences provided in S1 Table). The 3’ adaptors had a 6 nt randomized region at the 5’-end (Rand) or a 6 nt randomized region in the middle of the adaptor (MidRand). The 5’ adaptor had a 6 nt randomized region followed by a ‘C’ residue at the 3’-end (Rand). URef libraries were constructed and sequenced on the Ion PGM for each pair of adaptors shown in S4 Fig. The sequencing reads were annotated with a zero mismatch tolerance, counted, normalized such that each miRNA was expected to have a normalized read value of ‘1’, and then the data set was plotted as individual data points. The individual normalized values for each miRNA are available in S3 Table. It is clear from the graphs and the quantitative analysis that using a randomized adaptor in either ligation step results in a marked improvement in the sequencing results as indicated by more miRNAs being closer to their expected abundance, i.e. more RNAs <2-fold from the expected value and fewer RNAs >10-fold from the expected value. Surprisingly, the improvement in sequencing results was similar when either the 3’ Rand or 3’ MidRand adaptor were used. This is in contrast with the assumption that the bases at the 5’-end of the 3’ adaptor are the most important determinant of ligation efficiency [14]. The quantification improvement in sequencing results is greatest when a randomized adaptor is included in both ligation steps (3’Rand+5’Rand), where the number of RNAs <2-fold from the expected value is doubled while the number of RNAs >10-fold under the expected value is reduced by 5-fold relative to libraries made with 3’A1+5’A1 adaptors.

### miRNA ligation efficiency is improved when 5’ adaptors contain a region that is complementary to the target substrate

**Fig 4:**
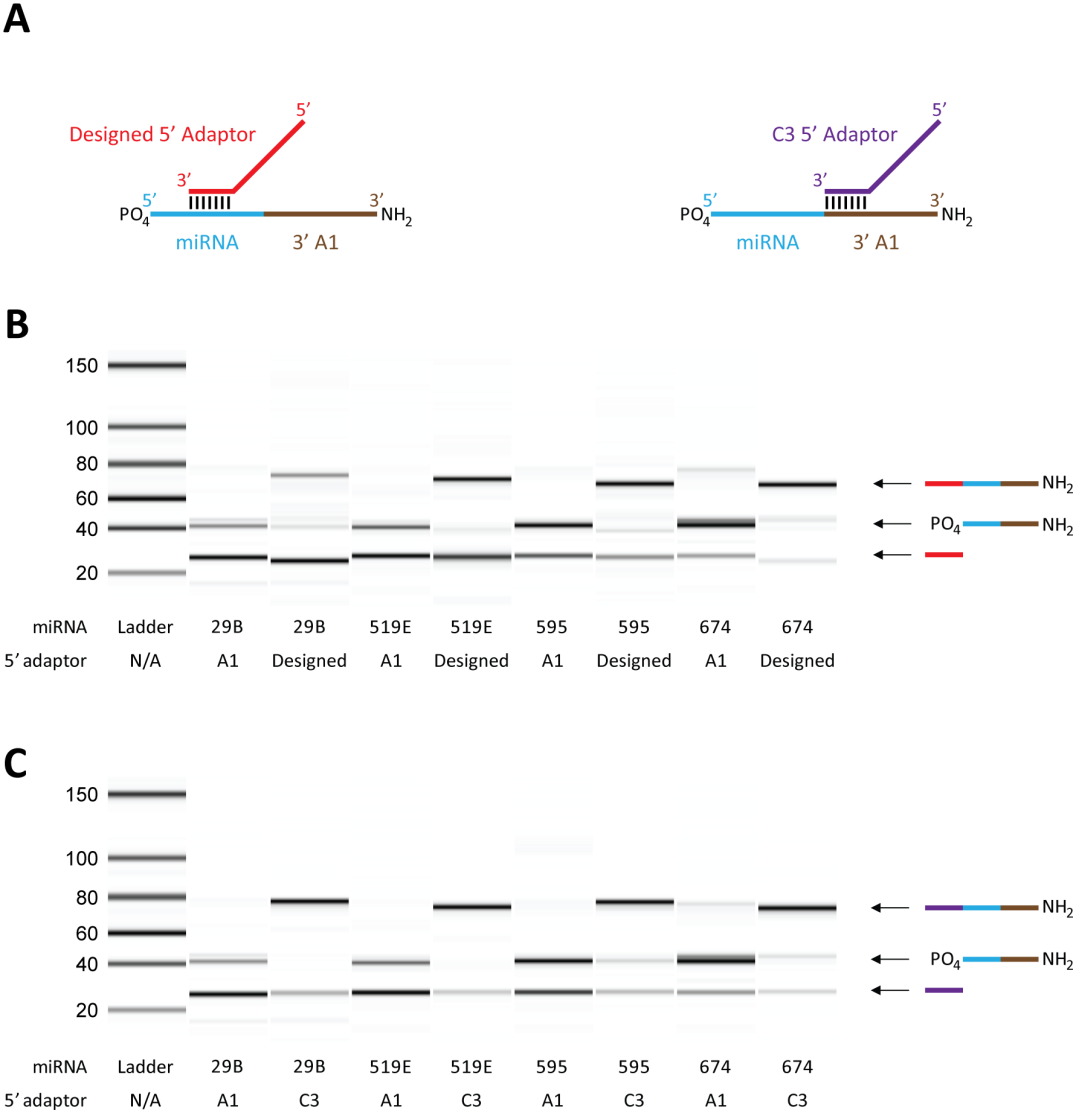
Improvement of 5’ adaptor ligation efficiency with designed adaptors. (A) Scheme depicting a miRNA (blue) attached to a 3’ A1 adaptor (brown) and designed 5’ adaptors that have a region complementary to the miRNA sequence (designed; red) or the 3’ adaptor sequence (C3; violet). (B,C) Comparison of 5’ ligation reaction products for four different miRNAs. Each ligation reaction contained a miRNA-3’ A1 hybrid and either the 5’ A1 adaptor, designed 5’ adaptor (B) or C3 5’ adaptor (C), ATP and T4 RNA ligase 1. The reaction products were resolved using an Agilent Small RNA kit and an Agilent 2100 Bioanalyzer (Agilent Technologies).

The results of our folding attribute analysis and randomized adaptor HTS experiments indicate that the cofold of miRNAs with adaptors have significant impact on HTS results. Considering this, we determined that in order to design adaptors that would reduce bias in library construction as well as increase ligation efficiency we should design adaptors that would result in a ‘favorable’ cofold. The observation that categories 9 and 12 in the 5’ cofold analysis of HTS results were enriched in highly represented miRNAs (Fig 3D) suggested that a miRNA would be efficiently ligated to a defined sequence adaptor with which it is capable of forming 5’ cofold structure 9 or 12. To test this idea, we chose 4 miRNAs (miR-29B, miR-519E, miR-595, miR-674) and subjected them to a 5’ ligation reaction with either the 5’ A1 adaptor or to an adaptor specifically designed to achieve cofold structure 9 with the miRNA (sequences are found in S1 Table, cofold of the 4 miRNAs with adaptors in S4 Table). The miRNA oligos used in the 5’ ligation experiments had the 3’ A1 adaptor sequence (DNA) included in order to mimic the molecule that would be produced after 3’ adaptor ligation (Fig 4A). The results of ligation experiments show that, after 1 hour at 25°C in the presence of T4 Rnl1 and ATP, the 4 miRNAs have virtually no detectable ligation product with the 5’ A1 adaptor, but are all efficiently ligated to their designed 5’ adaptors (Fig 4B). This observation supports the idea that the cofold data generated by this study can be used to design adaptors for specific miRNAs. However, it is unrealistic to design and include an adaptor for each miRNA in a complex pool of miRNAs, especially since the content of the pool is unknown in typical library construction experiments. To address this issue, we designed a 5’ adaptor where the last 7 bases on the 3’-end were complementary to the first 7 bases on the 5’-end of the 3’ A1 adaptor and called it the C3 adaptor (Fig 4A). The ability to pair with the 3’ adaptor sequence makes it more likely that cofold structures such as category 9 and 12 can form during 5’ adaptor ligation and therefore less likely that ‘unfavorable’ cofold structures such as category 8 and 16 can form (S5 Fig). Ligation experiments show that the 4 miRNAs that were tested in the designed adaptor experiments were also efficiently ligated to the C3 adaptor, which indicates the potential of the C3 adaptor for enhancing ligation efficiency (Fig 4C).

### Adaptors with internal randomized regions and a complementary region reduce bias by improving representation of RNA sequences that have ‘unfavorable’ ligation attributes

**Fig 5:**
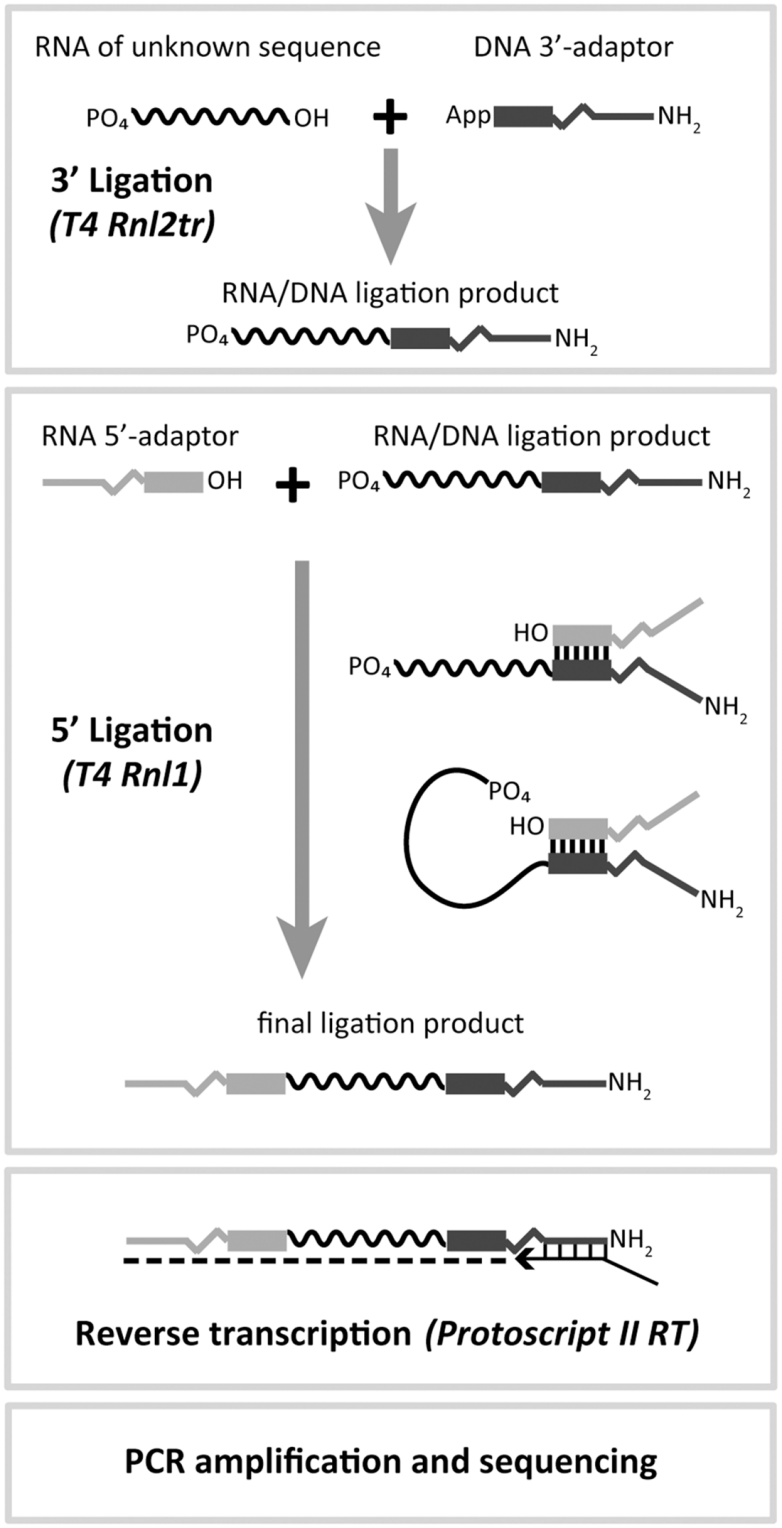
HTS library construction scheme for miRNAs using adaptors with randomized and complementary regions. An RNA sample is subjected to a ligation reaction to attach a 3’ adaptor. This reaction contains T4 RNA ligase 2, truncated (T4 Rnl2tr) or T4 RNA ligase 2, truncated KQ (T4 Rnl2trKQ) and an adenylated DNA adaptor that contains a 6 nt internal randomized region. Next, the natural presence of a phosphate group on the 5’-end of miRNAs is exploited as a substrate to ligate an RNA adaptor to the 5’-end in the presence of T4 RNA ligase 1 (T4 Rnl1) and ATP. The RNA adaptor used in the 5’ ligation step contains a 6 nt internal randomized region and a 7 nt region at its 3’ end which is complementary to 7 nt in the 3’ adaptor sequence. The defined sequence in the adaptors adjacent to the randomized regions allow the final ligation products to be reverse transcribed and PCR amplified in order to introduce the primer regions needed for HTS.

**Fig 6:**
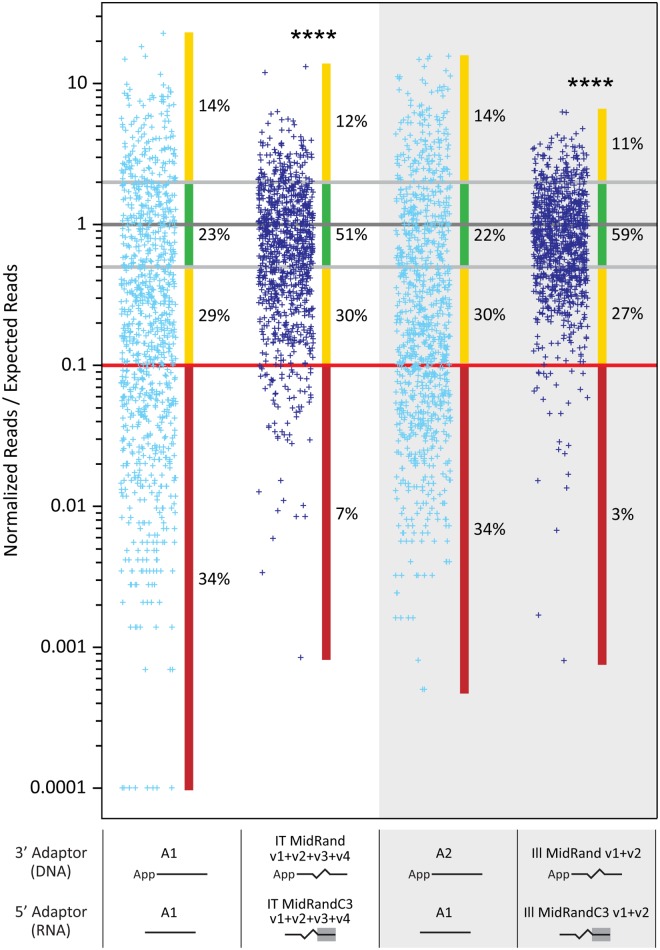
HTS results from libraries made with the miRXplore Universal Reference and new adaptors. The adaptors used in library construction are represented by solid lines (defined sequence), jagged lines (randomized sequence), and/or a solid line with a gray background (region that is complementary to the 5’-end of the 3’ adaptor) and their sequences can be found in S1 Table. Normalized reads for the 962 miRNA sequences in each data set are presented as individual data points on a logarithmic scale. Each miRNA was expected to have a normalized reads value of 1 (dark gray line). The interval of 2-fold from the expected value (equivalent to a 2-fold over or under representation) is shown with light gray lines and 10-fold under the expected value is shown with a red line. The percentage of data points that are >10-fold under the expected value is shown next to a vertical red bar, and the percentage <2-fold from the expected value is shown next to a vertical green bar. The percentages of data points in other regions are shown next to vertical yellow bars. The Mann-Whitney test was used to compare each data set to the 3’ A1 + 5’ A1 data set to determine the statistical significance of the difference between the two sets. Two-tailed p values are indicated as (****) p < 0.0001. The normalized read value for each miRNA in each data set can be found in S3 Table.

**Fig 7:**
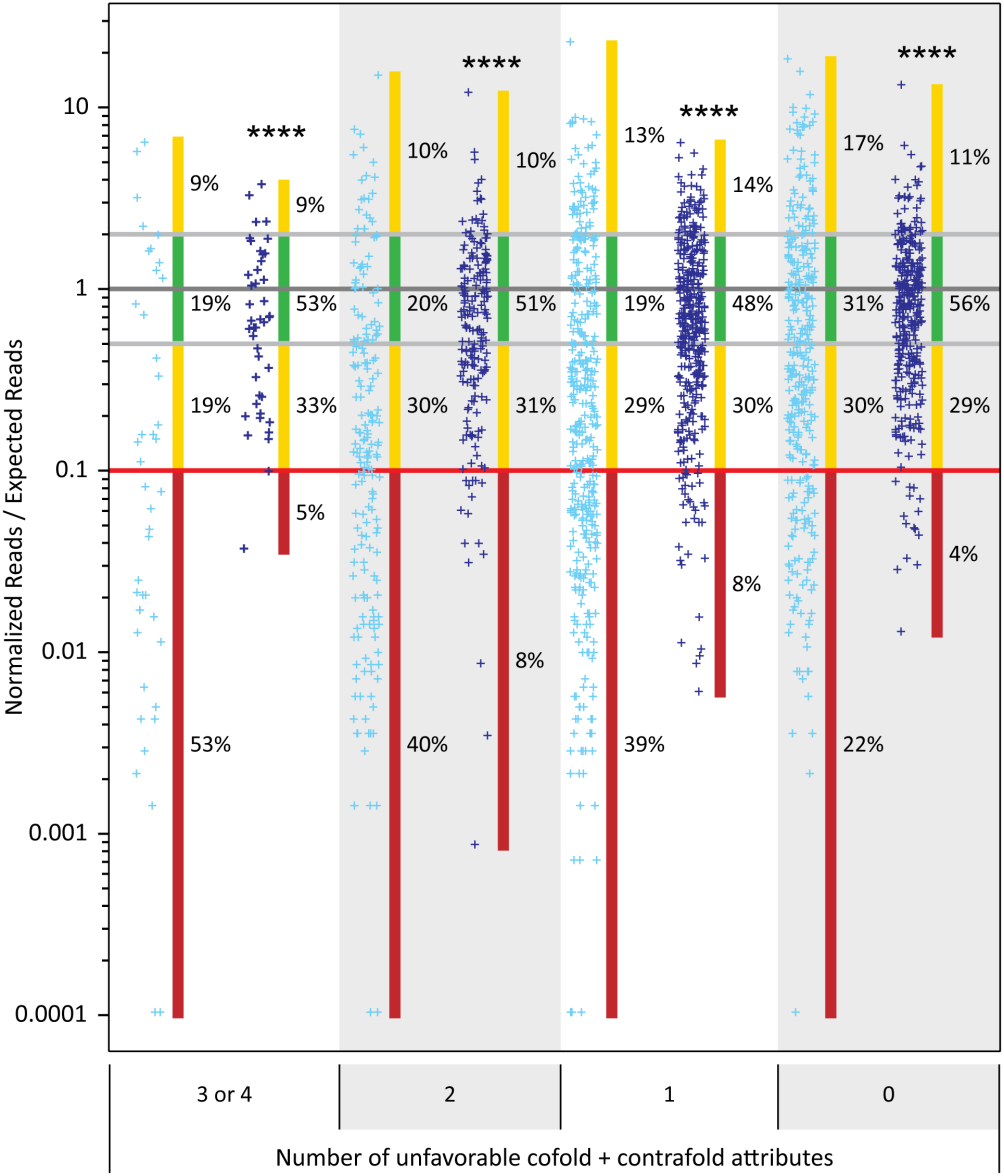
Relating the total number of unfavorable cofold and CONTRAfold attributes to miRNA sequence representation and improvement in HTS results. The HTS results for libraries constructed with the miRXplore Universal Reference and 3’ A1 + 5’ A1 or 3’ IT MidRand + 5’ IT MidRandC3 adaptors were separated into groups depending upon the number of “unfavorable” attributes for each miRNA sequence. The four attributes used to make the groups were the 3’ CONTRAfold and 5’ CONTRAfold of each miRNA and the 3’ cofold and 5’ cofold of each miRNA with the 3’ A1 and 5’ A1 adaptors, respectively. An “unfavorable” attribute was defined as having an enrichment value of -10% or worse as shown in our previous work (for 3’ CONTRAfold; [9]), S2 Fig panel C (5’ CONTRAfold), Fig 3B (3’ cofold), and Fig 3D (5’ cofold). Normalized reads for the miRNAs in each group are presented as individual data points on a logarithmic scale. Light blue points correspond to sequencing results with 3’ A1 + 5’ A1 adaptors and dark blue points correspond to sequencing results with 3’ IT MidRand + 5’ IT MidRandC3 adaptors. Each miRNA was expected to have a normalized reads value of 1 (dark gray line). The interval of 2-fold from the expected value (equivalent to a 2-fold over or under representation) is shown with light gray lines and 10-fold under the expected value is shown with a red line. The percentage of data points that are >10-fold under the expected value is shown next to a vertical red bar, and the percentage <2-fold from the expected value is shown next to a vertical green bar. The percentages of data points in other regions are shown next to vertical yellow bars. The Mann-Whitney test was used to compare each 3’ IT MidRand + 5’ IT MidRandC3 data set to its corresponding 3’ A1 + 5’ A1 data set to determine the statistical significance of the difference between the two sets. Two-tailed p values are indicated as (****) p < 0.0001.

Combining the observations from above, we designed new pairs of adaptors for use in library construction. These adaptors have an internal six base randomized region and a 7 base complementary region and were named 3’ MidRand and 5’ MidRandC3 (sequences in S1 Table). A schematic showing library construction with these adaptors is shown in Fig 5. We produced libraries from the URef RNA mix for sequencing on the IonPGM and Illumina MiSeq instruments to compare single sequence adaptors with these new adaptor combinations. Two sets of new adaptors (v1 + v2) were used for Illumina sequencing in order to generate sequence diversity for the instrument in the 7 base region between the randomized region and RNA insert and four sets were used for the IonPGM (v1+v2+v3+v4). The results show that the new adaptors produce a much more accurate representation of the URef mix as the sequencing reads cluster much more closely to the expected value than with single sequence adaptors (Fig 6). Specifically, the number of RNAs that were within 2-fold of their expected value increased from 23% to 51% in the IonPGM results and from 22% to 59% for the Illumina results. Perhaps more importantly, RNAs that were severely under-represented, which we define as being >10-fold under the expected value, decreased from 34% to 7% and 34% to 3%, respectively. In previous sections, we showed that 4 individual secondary structure and cofolding attributes influenced representation of RNA sequences in HTS results. We also wondered if the 4 attributes would contribute to a cumulative effect and would explain why so many sequences are under-represented in the single sequence adaptor libraries. To investigate this, each of the 962 unique miRNA sequences in the URef mix was placed into a group based on the total number of unfavorable attributes (0, 1, 2, 3 or 4) from cofold analysis and CONTRAfold analysis with the 3’ A1 and 5’ A1 adaptors (S5 Table). The IonPGM HTS results from libraries constructed with 3’ A1 + 5’ A1 adaptors and libraries constructed with 3’ MidRand + 5’ MidRandC3 adaptors were plotted for each group (Fig 7). The results show a trend where the sequencing reads for a given miRNA are more likely to be closer to the expected value when the miRNA sequence has fewer “unfavorable” cofold + CONTRAfold attributes with the A1 adaptors. For example, 53% of the sequences that have 3 or 4 of these attributes are >10-fold under-represented while only 22% of sequences with 0 of these attributes are >10-fold under-represented. Although there are outliers in each group, these results partially explain why certain miRNAs are under or over-represented when 3’ A1 + 5’ A1 adaptors are used to make libraries. The HTS data from 3’ IT MidRand + 5’ IT MidRandC3 adaptor libraries show that the miRNAs that had “unfavorable” attributes with the A1 adaptors now have improved representation. For instance, the group of RNAs with 3 or 4 “unfavorable” attributes with the A1 adaptors only have 5% of the RNAs >10-fold under-represented and have 53% within 2-fold of the expected value in the sequencing results generated by the MidRand adaptors. This suggests that the randomized adaptors mitigate the effects of bias introduced by RNA secondary structure and RNA-adaptor interactions by providing a variety of adaptor folding options for each RNA.

**Fig 8:**
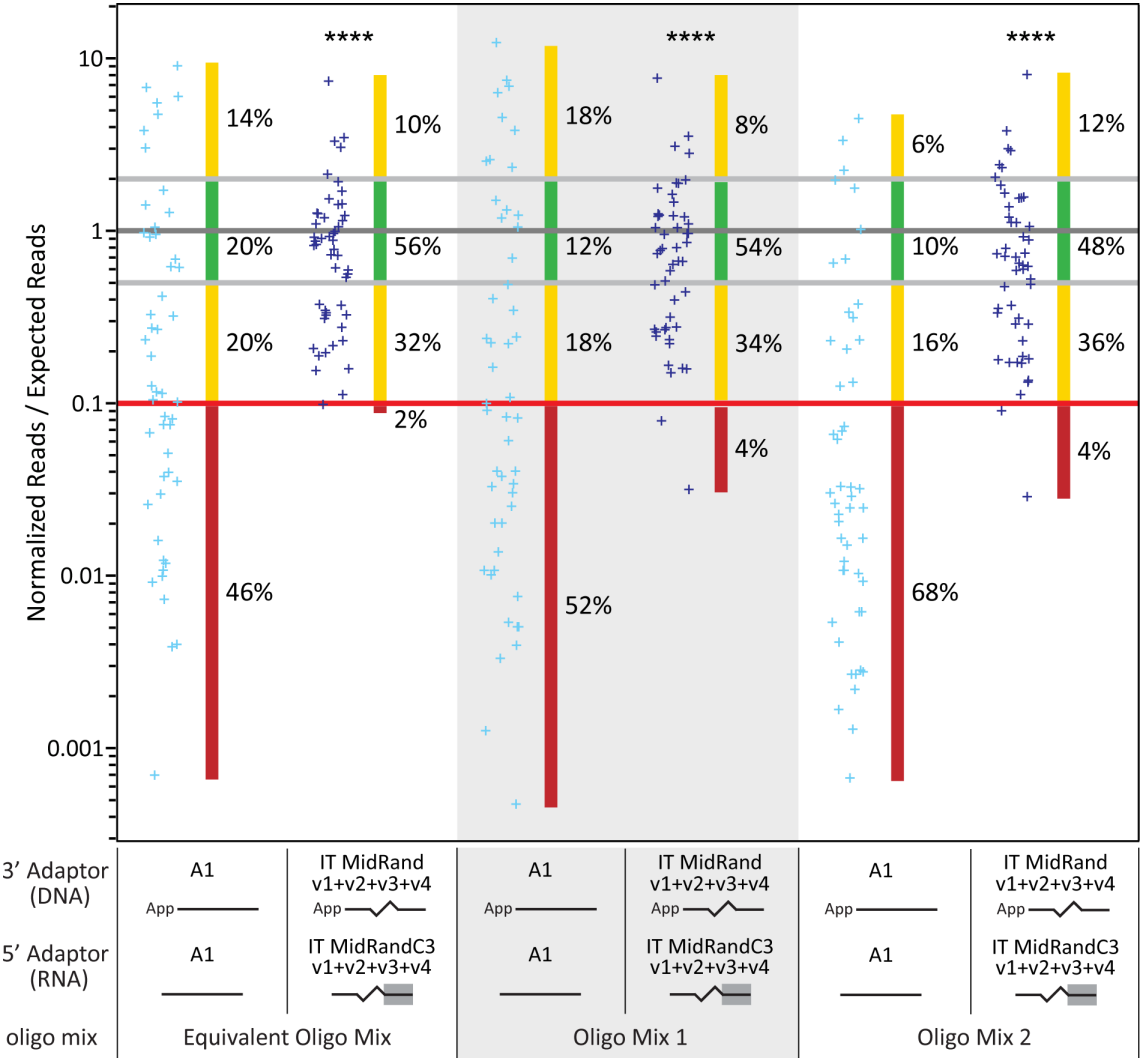
HTS results from libraries made using different oligonucleotide mixtures. Three oligonucleotide mixtures were subjected to HTS using different adaptors, as indicated. The adaptors used in library construction are represented by solid lines (defined sequence), jagged lines (randomized sequence), and a solid line with a gray background (region that is complementary to the 5’-end of the 3’ adaptor) and their sequences can be found in S1 Table. These oligonucleotide mixtures each contained the same 50 sequences, but the sequences were mixed in different amounts; either equivalent or different 500-fold spreads in concentration (S6 Table). Normalized reads for the 50 miRNA sequences in each data set are presented as individual data points on a logarithmic scale. Each miRNA’s normalized reads value was divided by its expected value given the concentration of the miRNA in the defined mix. A result of “1” after this division indicates that the normalized reads were equal to the expected value (dark gray line). The interval of 2-fold from the expected value (equivalent to a 2-fold over or under representation) is shown with light gray lines and 10-fold under the expected value is shown with a red line. The percentage of data points that are >10-fold under the expected value is shown next to a vertical red bar, and the percentage <2-fold from the expected value is shown next to a vertical green bar. The percentages of data points in other regions are shown next to vertical yellow bars. The Mann-Whitney test was used to compare each IT MidRand data set to its corresponding A1 adaptor data set to determine the statistical significance of the difference between the two sets. Two-tailed p values are indicated as (****) p < 0.0001. The values used to make the graph can be found in S6 Table.

While our new adaptor designs produced good results when using the URef mix as a substrate, we wanted to confirm their usefulness on other substrates. Specifically, the URef mix consists of 962 miRNA sequences mixed in equal abundance, but we wondered how the adaptors would perform when a mix of miRNA sequences in different concentrations were used. We ordered 50 RNA oligonucleotides corresponding to miRNA sequences and made three defined mixtures of them (S6 Table). In one mix the 50 sequences were in equal concentration, in another there was a 500-fold spread in concentration from the most abundant to least abundant, and in the third mix there was also a 500-fold spread, but the order of which sequences were high or low differed from the second mix. These mixes were subjected to library construction and sequencing on the IonPGM using either the A1 adaptors or the 3’ IT MidRand + 5’ IT MidRandC3 adaptors. The results for all three oligonucleotide mixtures show that the new adaptors produce results that are much more representative of the input RNA mixture than the A1 adaptors (Fig 8). Not only is there a significant increase in amount of RNAs within 2-fold of their expected value, an average of 14% for A1 versus 52% for new adaptors, but there is also a dramatic reduction in the amount of RNAs that are >10-fold under represented, an average of 55% for A1 versus 3% for new adaptors. These results demonstrate the utility of our new adaptor designs in producing more accurate results for both abundant and scarce sequences in an RNA sample.

## Discussion

Compared to low-throughput techniques, HTS offers many benefits for sRNA research such as the ability to detect the entire sRNA population in a sample in a single experiment, including previously unknown sRNAs. HTS also has advantages over other high-throughput techniques that rely on a hybridization-based method such as microarray analysis. These advantages include better sensitivity of detection and the ability to determine sequence at single nucleotide resolution, which allows accurate identification of closely related sRNA species and the identification of putative RNA editing events. Unfortunately, there is also a disadvantage for using HTS, which is that the sequencing reads generated by HTS do not accurately reflect the relative quantity of sRNAs in a sample [13–15]. Several recent studies have determined that this disadvantage is the result of bias introduced by the ligation steps during sRNA library construction. In one such study, Hafner et al. used pools of 770 and 815 miRNAs to investigate several of the variables in sRNA library construction [13]. They concluded that the ligation steps had the largest contribution to the bias and that the structural properties of miRNAs and adaptors were responsible for differences in ligation efficiency. However, they did not investigate a solution to the ligation bias problem, nor perform an exhaustive investigation of miRNA-adaptor structural properties. Jayaprakash et al. prepared sRNA libraries from total RNA with either defined sequence adaptors or adaptors containing either 2 or 4 randomized nucleotides adjacent to the ligation junction [14]. They also concluded that ligation efficiency is the primary contributor to bias in HTS results and that the T4 RNA ligases have primary sequence biases that are the cause of differences in ligation efficiency. Unfortunately, the lack of any secondary structure analysis limits the scope of these results. Sorefan et al. used a 21 nt randomized RNA oligonucleotide pool as a substrate for library construction [15]. They observed that if adaptors with a 4 nt randomized region at the ligation junction (HD adaptors) were used in library construction, then there was an increase in complexity of the sequencing reads that they obtained. This increase in complexity was also seen if they used a biological RNA sample and their HD adaptors. In both cases, the composition of the starting material was not well defined which makes comparison of miRNA abundance in the HTS results to miRNA abundance in the sample very difficult. In our recent study, we investigated ligation bias by using a pool of randomized RNA oligonucleotides and the 3’ adaptor ligation step in isolation [9]. We concluded that adaptors containing randomized regions help mitigate ligation bias and that this is due to structural characteristics of the RNAs and adaptors, not primary sequence. All of the studies described above suffer from one or more caveats that limit the usefulness of the results towards achieving a better sRNA library construction method. The most important caveat is that none of the studies used a complex, defined RNA sample, subjected it to library construction with defined sequence versus randomized adaptors, and then compared the HTS results to the expected results given that the starting material was defined. In this study we sought to use a complex, defined sample and various combinations of adaptors in order to conduct the most comprehensive analysis of sRNA HTS library construction to date.

In order to have more information about T4 RNA ligase substrate preferences prior to conducting HTS experiments on a defined sample, we first investigated the 5’ adaptor ligation step in isolation. We used an approach where a pool of oligos were synthesized that consisted of a 21 nt randomized region and an attached 3’ A1 adaptor sequence. The fact that the 3’ adaptor sequence was already built into the oligonucleotides allowed us to start library construction at the 5’ adaptor ligation step using T4 Rnl1 and assess the output of HTS sequencing without the influence of a 3’ adaptor ligation step. Also, an aliquot of the randomized oligonucleotide was taken and synthesis was allowed to continue through to include the entire 5’ A1 sequence. This allowed us to make a library for sequencing that was independent of ligation altogether and the HTS sequencing data from this library could be used to assess the input of the randomized oligonucleotide pool. Our conclusions from this approach were that there was minimal primary sequence bias in the ligated libraries, RNAs with secondary structure that included a paired base at the ligation site were depleted in ligated libraries, and that the cofold structure between the RNA and adaptor influenced the representation of RNAs in the ligated libraries. These conclusions are consistent with our previous study where we investigated 3’ adaptor ligation in isolation [9]. There are differences in the two studies between which specific cofold structure classes are depleted or enriched in ligated libraries. We do not find this surprising considering that the substrates of the two ligations are different, an RNA plus an adenylated DNA adaptor for the 3’ adaptor ligation and an RNA plus an unmodified RNA adaptor for the 5’ adaptor ligation. Overall, the 5’ adaptor ligation data supported the conclusion that the main contributor to ligation bias is not at the primary sequence level and is instead at the level structure within and between RNAs and adaptors.

For our sRNA library construction experiments on defined samples, we used the miRXplore Universal Reference (URef) and custom oligonucleotide mixes. The power of using these samples for studying the results of HTS experiments is that the expected concentration of each miRNA sequence is known and is not an additional variable as it is with a biological sample of unknown composition. The results of libraries constructed with single sequence adaptors show that the relative quantity of miRNAs according to number of sequencing reads does not cluster densely around their expected value. When we used adaptors that contained randomized regions of 6 nt in length we obtained HTS results that cluster more densely around the expected value. This was true whether the randomized region started at the ligation junction or was in the middle of the adaptor. Randomized adaptor regions adjacent to the ligation junctions can further complicate sequence analysis if an unknown sample were to be sequenced because the termini of the sRNA in a sequencing read cannot be precisely determined. For this reason, we chose to use adaptors with internal random regions for the remainder of the study. Improvement in HTS results regardless of the position of the randomized sequence within the adaptors indicates that the sequence flanking the ligation junction is not the most important determinant of ligation efficiency, which is in contrast to a previous study [14]. The fact that changing the base composition of the middle of the adaptor significantly affects HTS results also suggests that using barcoded adaptors during ligation so that different samples can be sequenced in the same sequencing run is a risky approach if the intent of the experiment is to quantify and compare the expression profile of the different samples. Due to this, we recommend that barcodes only be introduced in either the primer used for reverse transcription, or better yet, in the PCR primers. We also observed more accurate/less biased HTS results when a 5’ adaptor with a randomized region and/or a region complementary to the 3’ adaptor was used, but even better results were seen when both of the single sequence 3’ and 5’ adaptors were substituted with randomized and complementary adaptors. Although our new adaptors do not produce HTS results where all miRNA sequences are represented at their expected number of sequencing reads, the results are clearly a marked improvement versus using single sequence adaptors.

In addition to demonstrating the impact that ligation bias has on HTS results, experiments with the URef miRNA pool generated data that shed light on which factors have the most influence over whether a particular miRNA will end up under-represented or over-represented in HTS results. Our results show that while primary sequence may contribute a small amount to ligation bias and that secondary structure within the miRNA itself makes a contribution, it is the interaction between the adaptor and miRNA that has the most influence for both ligation steps. A cofold between the miRNA and adaptor that looks like a simple stem loop with the ligation junction in the loop appears to be the most preferred interaction, while structures where the ligation junction is located a bulged portion of a stem tend to be unfavorable. Care must be taken when analyzing these structures because the cofold analysis represents only the most stable interaction between a particular miRNA and adaptor and there are potentially many possible structures that could be formed that are slightly less stable yet present because of the dynamic nature of a sample in solution. Our results with complementary adaptors indicate that increasing the strength of the adaptor-substrate interaction can increase ligation efficiency for some miRNAs even if the most stable cofold structure is one that is considered unfavorable for ligation. This suggests that cofold structure as well as the strength of the miRNA-adaptor interaction are both factors in ligation efficiency, additional studies may determine which is ultimately the most important.

While the quantification bias is unlikely to cause problems when assessing sRNA expression differences between samples that were subjected to library construction with the same adaptors and protocol, there are many instances where HTS bias is a large problem. As noted in other publications, inaccurate quantification can potentially cause the incorrect annotation of the star strand versus active strand of a miRNA, it can also make the identification of which RNAs warrant additional analysis difficult since it is unclear which RNAs are actually highly expressed in the sample, and it makes the comparison of HTS results from libraries made with different adaptors very difficult if not impossible [10,11,13,15,26]. Although the randomized and complementary adaptor approach is clearly an improvement over standard methods, the elimination of bias during library construction should be a major goal for the future. This will hopefully be achieved through a combination of more adaptor design breakthroughs and continued improvements in protocol and ligation optimization. The ability to generate completely unbiased HTS results would make HTS into an even more powerful gold standard for most aspects of sRNA research.

## Supporting Information

S1 Fig Library construction scheme for studying 5’ adaptor ligation. A mix of RNA oligonucleotides consisting of a 21 nt defined sequence (solid line) and a 21 nt randomized sequence (jagged line) were ligated to the 5’ A1 adaptor using T4 RNA ligase 1 (T4 Rnl1). The ligation products were reverse transcribed and PCR amplified in order to introduce the primer regions needed for Ion PGM sequencing. Sequences of all oligonucleotides can be found in S1 Table.
(TIF)

S2 Fig Summary of the sequencing data generated from the randomized RNA oligonucleotides approach. (A) The nucleotide frequency at each position in the 21 nt randomized region. The frequencies were calculated for the input (full length synthesized RNA oligonucleotides) and output (ligated RNA oligonucleotides) and then plotted in enoLOGOS format [27]. The frequency of each nucleotide is proportional to the height of its corresponding letter. (B) Enrichment of a particular nucleotide at a given position in the output versus the input. The values plotted are the normalized nucleotide frequency percentage (RNnp)– 25%. If RNnp—25% of a nucleotide at a given position is equal to 0, it indicates that there is no preference for the nucleotide at that position. If RNnp—25% is greater to or less than 0, that indicates that the nucleotide is preferred or not preferred, respectively. (C) Enrichment of RNAs with secondary structure at the 5’-end. Every sequence in the input and output was categorized based on the number of unpaired nucleotides at the 5’-end as predicted by CONTRAfold analysis [23]. The value of enrichment was determined by the equation ‘(output-input)/input’. A positive enrichment value indicates that the category is enriched in the output, while a negative enrichment value indicates that the category is enriched in the input. (D) Definition of cofold structure categories. Cofold analysis between two nucleic acid ligation substrates was carried out with Vienna RNAcofold software [29]. Cofold results were separated into 16 categories based on the possible combinations of free nucleotides (dots) and paired nucleotides (brackets) around the ligation junction (represented by ‘&’). The directionality of the brackets indicates the pairing orientation. Generalized diagrams of corresponding cofold structures are shown under the dot-bracket notation. The molecule on the 5’ side of the ligation junction is shown in red and the molecule on the 3’ side of the junction is shown in black. (E) Distribution of cofold categories when the 5’ A1 adaptor sequence was cofolded with all sequences from the randomized oligonucleotides present in the input (red) and output (blue) data sets. (F) Enrichment of cofold categories in the output versus the input. The enrichment was calculated using the same equation as in (C).
(TIF)

S3 Fig Relating individual cofold and CONTRAfold attributes to miRNA representation in HTS results. The sequencing data for libraries constructed with the miRXplore Universal Reference and A1 adaptors were separated into groups depending upon which miRNAs have “unfavorable” or “favorable” CONTRAfold or cofold attributes with 3’ A1 + 5’ A1 adaptors. An attribute was defined as “unfavorable” if its category had an enrichment value of -10% or worse and “favorable” if the enrichment value was at least +5% for 3’ CONTRAfold and 5’ CONTRAfold or +20% for 3’ cofold and 5’ cofold, as defined in our previous work (for 3’ CONTRAfold; [9]), S2 Fig, panel C (5’ CONTRAfold), Fig 3B (3’ cofold), and Fig 3D (5’ cofold). Normalized reads for the miRNAs in each group are presented as individual data points on a logarithmic scale. Each miRNA was expected to have a normalized reads value of 1 (dark gray line). The interval of 2-fold from the expected value (equivalent to a 2-fold over or under representation) is shown with light gray lines and 10-fold under the expected value is shown with a red line. The percentage of data points that are >10-fold under the expected value is shown next to a vertical red bar, and the percentage <2-fold from the expected value is shown next to a vertical green bar. The percentages of data points in other regions are shown next to vertical yellow bars. The Mann-Whitney test was used to compare each ‘favorable’ data set to its corresponding ‘unfavorable’ data set to determine the statistical significance of the difference between the two sets. Two-tailed p values are indicated as; (ns) p > 0.05, (****) p < 0.0001.
(TIF)

S4 Fig HTS results of libraries made from the miRXplore Universal Reference with different combinations of adaptors. The adaptors used in library construction are represented by solid lines (defined sequence), jagged lines (randomized sequence), and a solid line with a gray background (region that is complementary to the 5’-end of the 3’ adaptor) and their sequences can be found in S1 Table. The normalized reads for the 962 miRNA sequences in each data set are presented as individual data points on a logarithmic scale. Each miRNA was expected to have a normalized reads value of 1 (dark gray line). The interval of 2-fold from the expected value (equivalent to a 2-fold over or under representation) is shown with light gray lines and 10-fold under the expected value is shown with a red line. The percentage of data points that are >10-fold under the expected value is shown next to a vertical red bar, and the percentage <2-fold from the expected value is shown next to a vertical green bar. The percentages of data points in other regions are shown next to vertical yellow bars. The Mann-Whitney test was used to compare each data set to the 3’ A1 + 5’ A1 data set to determine the statistical significance of the difference between the two sets. Two-tailed p values are indicated as; (***) p < 0.001, (****) p < 0.0001. The normalized reads value for each miRNA in each data set can be found in S3 Table.
(TIF)

S5 Fig Distribution of cofold structures with the 5’ A1 and 5’ C3 adaptors for miRNAs in the miRXplore Universal Reference. All 962 miRNA sequences were cofolded with the 5’ A1 adaptor (red) or the 5’ C3 adaptor (blue) and the distribution by cofold category is shown.
(TIF)

S1 Table Oligonucleotide and adaptor sequences. (XLSX)

S2 Table Primary sequence composition at every position in randomized region of the ‘input’ and ‘output’ HTS data sets in the 5’ ligation study. (XLSX)

S3 Table Sequence and data summary for each miRNA in the miRXplore Universal Reference. The attributes in columns ‘E, F, G, H’ are color coded red, yellow, or green for ‘unfavorable’, ‘neutral’, or ‘favorable’ category, respectively as determined by our cofold and CONTRAfold results. For the 5’ cofold and 5’ CONTRAfold analysis, the 3’ A1 adaptor sequence was added to the 3’ end of the miRNA sequence. The data in columns ‘I’ through ‘S’ are the average normalized reads values for each miRNA sequence in HTS results from libraries constructed with the adaptors listed at the top of the column (adaptor sequences can be found in S1 Table) and each data value is color coded red (greater than 10-fold under expected value), green (within 2-fold of expected value), or yellow (other). The experiments whose data are represented in columns “P” through “S” were performed with a different lot of the miRXplore Universal Reference than what was used to generate the data in columns “I” through “O”.
(XLSX)

S4 Table 5’ cofold analysis of 4 miRNAs with different adaptors. The 3’ A1 adaptor sequence was added to the 3’ end of the miRNA sequence prior to cofolding with the 5’ adaptor. The adaptor sequences can be found in S1 Table.
(XLSX)

S5 Table HTS results from miRXplore Universal Reference libraries broken into groups based on the number of “unfavorable” cofold and CONTRAfold attributes. The normalized reads values from columns “R” and “S” in S3 Table were used to make the graph in Fig 7 after the miRNA sequences were separated based on whether they had 0, 1, 2, 3 or 4 “unfavorable” (indicated by red) attributes when cofold or CONTRAfold analysis was carried out with the A1 adaptor sequences.
(XLSX)

S6 Table Composition of and normalized reads values for custom RNA oligonucleotide mixtures. Each mix contained all 50 of the sequences indicated at the concentrations shown. HTS results were generated by the IonPGM after library construction with the indicated adapters.
(XLSX)
