# Supplementary figures and images for: Bias in Ligation-Based Small RNA Sequencing Library Construction Is Determined by Adaptor and RNA Structure
Source: PLoS One. 2015 May 5;10(5):e0126049. doi: 10.1371/journal.pone.0126049 (PMC4420488; doi:10.1371/journal.pone.0126049)

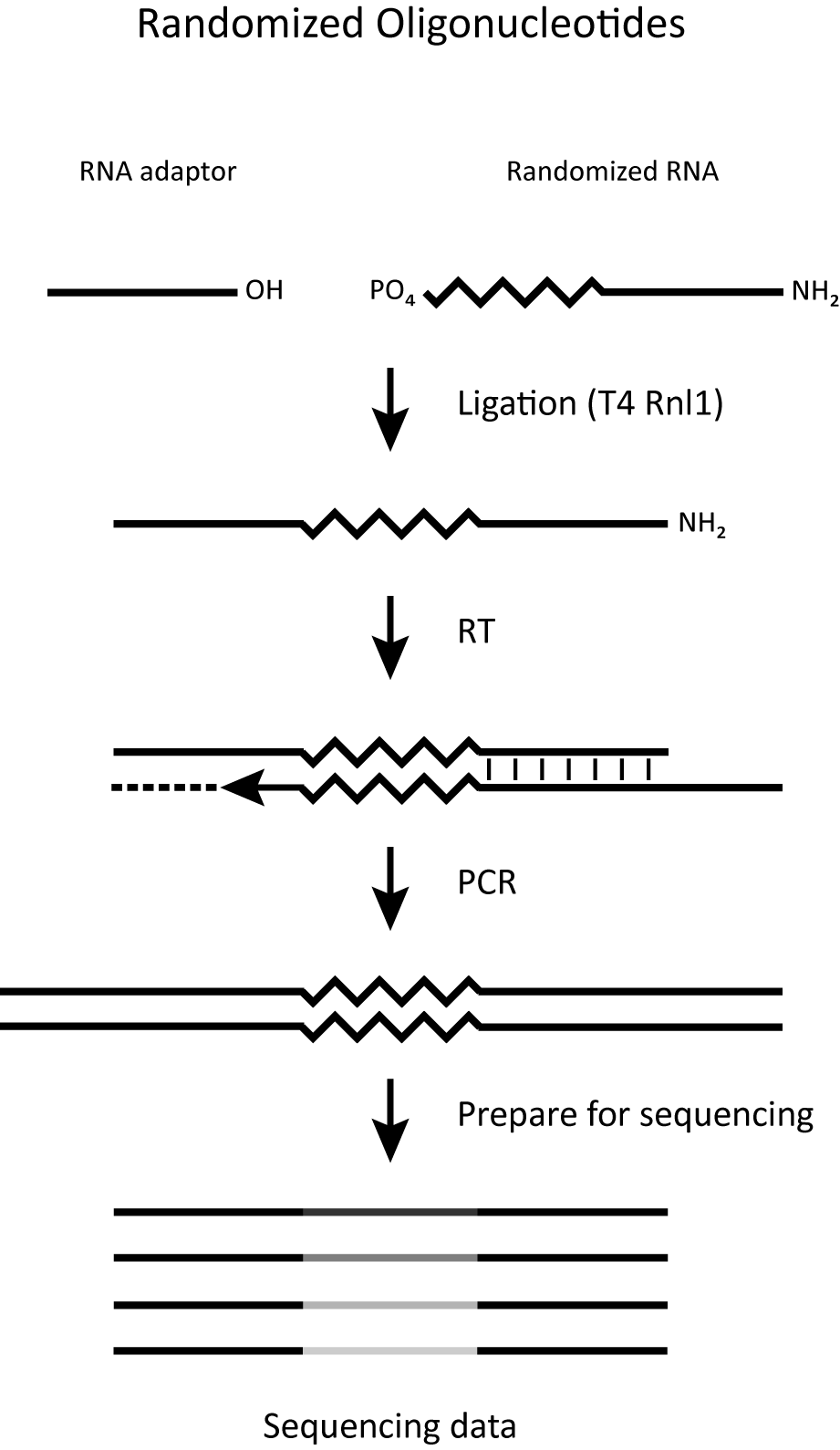

Supplement: S1 Fig — A mix of RNA oligonucleotides consisting of a 21 nt defined sequence (solid line) and a 21 nt randomized sequence (jagged line) were ligated to the 5’ A1 adaptor using T4 RNA ligase 1 (T4 Rnl1). The ligation products were reverse transcribed and PCR amplified in order to introduce the primer regions needed for Ion PGM sequencing. Sequences of all oligonucleotides can be found in S1 Table. (TIF) [file pone.0126049.s001.tif]

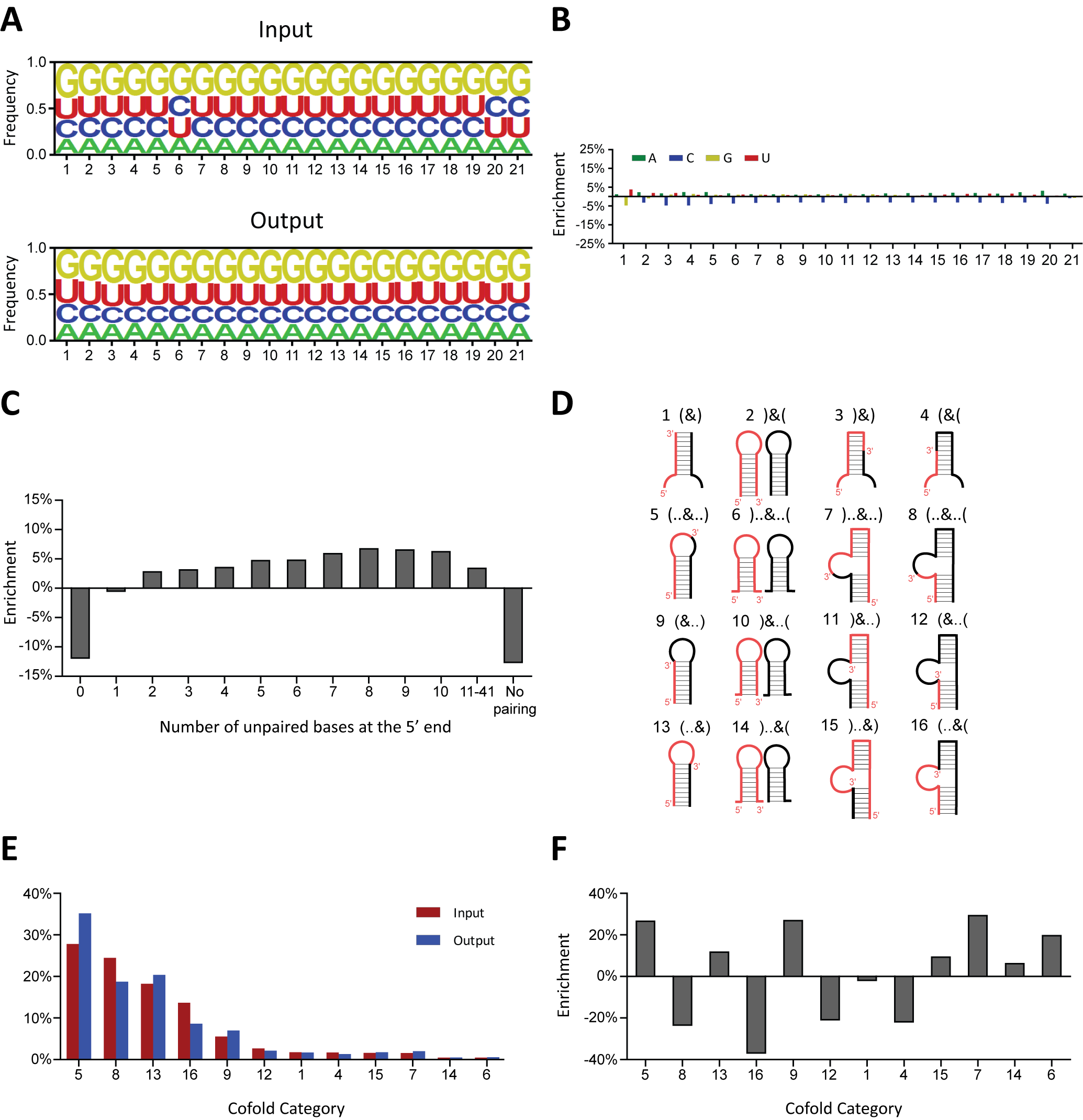

Supplement: S2 Fig — (A) The nucleotide frequency at each position in the 21 nt randomized region. The frequencies were calculated for the input (full length synthesized RNA oligonucleotides) and output (ligated RNA oligonucleotides) and then plotted in enoLOGOS format [27]. The frequency of each nucleotide is proportional to the height of its corresponding letter. (B) Enrichment of a particular nucleotide at a given position in the output versus the input. The values plotted are the normalized nucleotide frequency percentage (RNnp)– 25%. If RNnp—25% of a nucleotide at a given position is equal to 0, it indicates that there is no preference for the nucleotide at that position. If RNnp—25% is greater to or less than 0, that indicates that the nucleotide is preferred or not preferred, respectively. (C) Enrichment of RNAs with secondary structure at the 5’-end. Every sequence in the input and output was categorized based on the number of unpaired nucleotides at the 5’-end as predicted by CONTRAfold analysis [23]. The value of enrichment was determined by the equation ‘(output-input)/input’. A positive enrichment value indicates that the category is enriched in the output, while a negative enrichment value indicates that the category is enriched in the input. (D) Definition of cofold structure categories. Cofold analysis between two nucleic acid ligation substrates was carried out with Vienna RNAcofold software [29]. Cofold results were separated into 16 categories based on the possible combinations of free nucleotides (dots) and paired nucleotides (brackets) around the ligation junction (represented by ‘&’). The directionality of the brackets indicates the pairing orientation. Generalized diagrams of corresponding cofold structures are shown under the dot-bracket notation. The molecule on the 5’ side of the ligation junction is shown in red and the molecule on the 3’ side of the junction is shown in black. (E) Distribution of cofold categories when the 5’ A1 adaptor sequence was cofolded w [file pone.0126049.s002.tif]

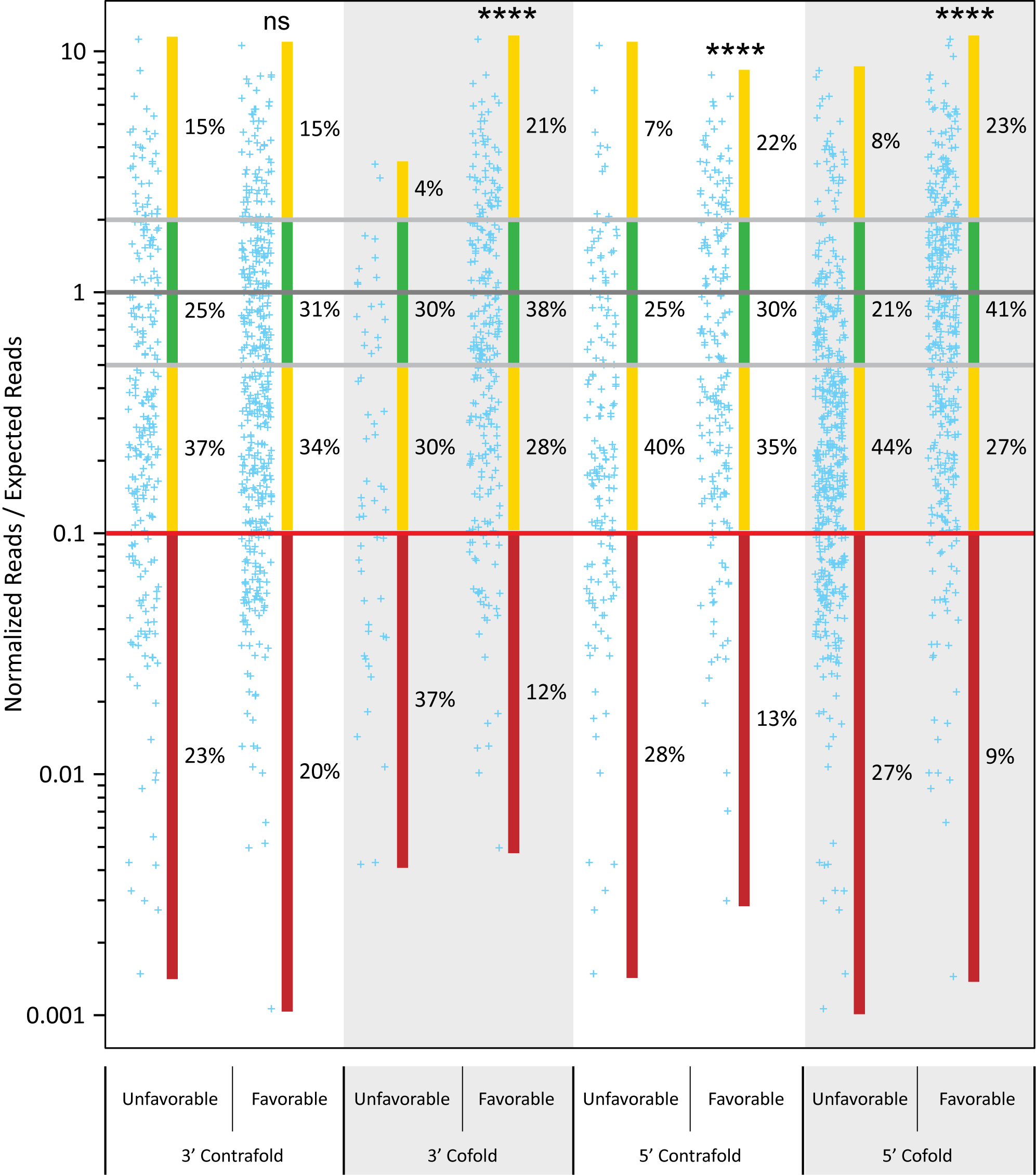

Supplement: S3 Fig — The sequencing data for libraries constructed with the miRXplore Universal Reference and A1 adaptors were separated into groups depending upon which miRNAs have “unfavorable” or “favorable” CONTRAfold or cofold attributes with 3’ A1 + 5’ A1 adaptors. An attribute was defined as “unfavorable” if its category had an enrichment value of -10% or worse and “favorable” if the enrichment value was at least +5% for 3’ CONTRAfold and 5’ CONTRAfold or +20% for 3’ cofold and 5’ cofold, as defined in our previous work (for 3’ CONTRAfold; [9]), S2 Fig, panel C (5’ CONTRAfold), Fig 3B (3’ cofold), and Fig 3D (5’ cofold). Normalized reads for the miRNAs in each group are presented as individual data points on a logarithmic scale. Each miRNA was expected to have a normalized reads value of 1 (dark gray line). The interval of 2-fold from the expected value (equivalent to a 2-fold over or under representation) is shown with light gray lines and 10-fold under the expected value is shown with a red line. The percentage of data points that are >10-fold under the expected value is shown next to a vertical red bar, and the percentage <2-fold from the expected value is shown next to a vertical green bar. The percentages of data points in other regions are shown next to vertical yellow bars. The Mann-Whitney test was used to compare each ‘favorable’ data set to its corresponding ‘unfavorable’ data set to determine the statistical significance of the difference between the two sets. Two-tailed p values are indicated as; (ns) p > 0.05, (****) p < 0.0001. (TIF) [file pone.0126049.s003.tif]

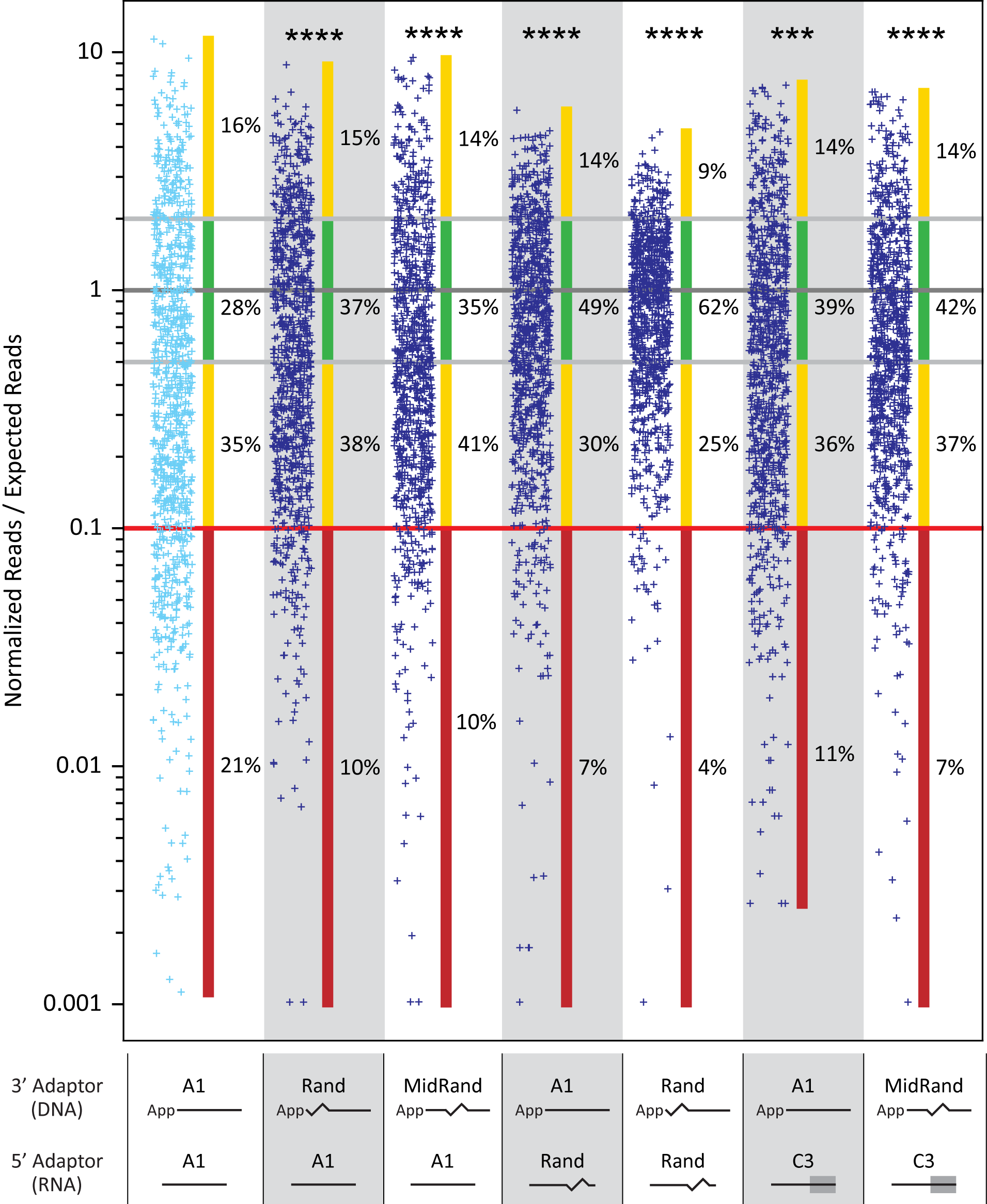

Supplement: S4 Fig — The adaptors used in library construction are represented by solid lines (defined sequence), jagged lines (randomized sequence), and a solid line with a gray background (region that is complementary to the 5’-end of the 3’ adaptor) and their sequences can be found in S1 Table. The normalized reads for the 962 miRNA sequences in each data set are presented as individual data points on a logarithmic scale. Each miRNA was expected to have a normalized reads value of 1 (dark gray line). The interval of 2-fold from the expected value (equivalent to a 2-fold over or under representation) is shown with light gray lines and 10-fold under the expected value is shown with a red line. The percentage of data points that are >10-fold under the expected value is shown next to a vertical red bar, and the percentage <2-fold from the expected value is shown next to a vertical green bar. The percentages of data points in other regions are shown next to vertical yellow bars. The Mann-Whitney test was used to compare each data set to the 3’ A1 + 5’ A1 data set to determine the statistical significance of the difference between the two sets. Two-tailed p values are indicated as; (***) p < 0.001, (****) p < 0.0001. The normalized reads value for each miRNA in each data set can be found in S3 Table. (TIF) [file pone.0126049.s004.tif]

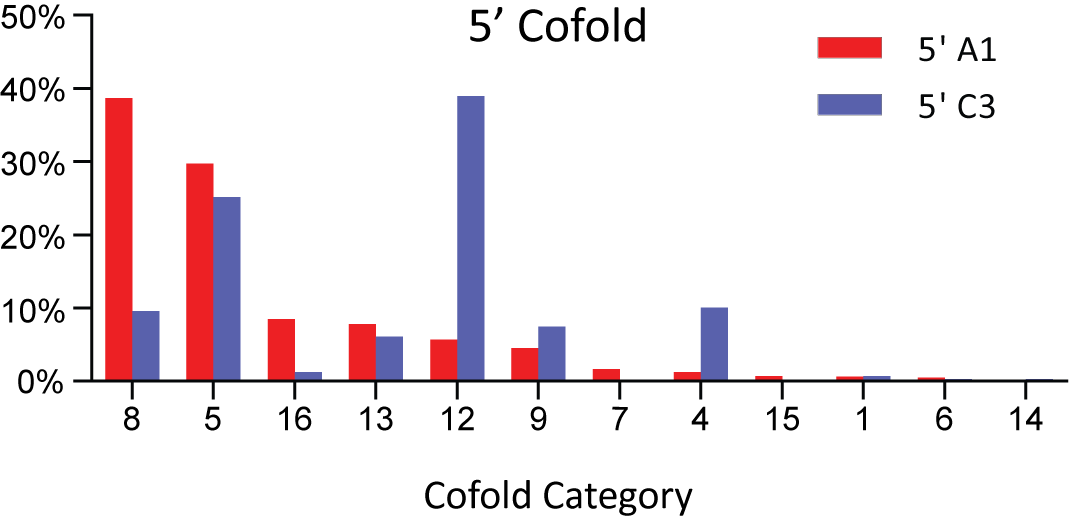

Supplement: S5 Fig — All 962 miRNA sequences were cofolded with the 5’ A1 adaptor (red) or the 5’ C3 adaptor (blue) and the distribution by cofold category is shown. (TIF) [file pone.0126049.s005.tif]
